# Enhancement of power quality in grid-connected systems using a predictive direct power controlled based PV-interfaced with multilevel inverter shunt active power filter

**DOI:** 10.1038/s41598-025-92693-3

**Published:** 2025-03-07

**Authors:** Rajendran Boopathi, Vairavasundaram Indragandhi

**Affiliations:** School of Electrical Engineering, Vellore Institute of Technology, Vellore, 632014 India

**Keywords:** Power quality, Photovoltaic array, Neutral-Point clamped inverter, Shunt active power filter, Predictive direct power control, Enhanced INC MPPT technique., Electrical and electronic engineering, Energy grids and networks

## Abstract

The integration of Nonlinear Loads (NLs) in industrial, commercial and residential settings over the past two decades has significantly worsened power quality issues in modern electrical distribution networks. In today’s modern era, the growing use of sensitive and expensive electronic devices makes it crucial to ensure power quality for the reliable and secure functioning of the power system. Shunt Active Power Filters (SAPF) are necessary to prevent current distortions caused by NLs from entering the grid. Otherwise, system effectiveness and power transmission capabilities would be diminished. In this work, we introduce a novel Predictive Direct Power Control (PDPC) strategy incorporating generating reference signals for SAPF model of a Three-level (3 L) Neutral-Point Clamped (NPC) inverter. This innovative system serves as a SAPF, specifically designed to attenuate the harmonics emerging from abrupt increments in NLs. Moreover, it proactively addresses the challenge of reactive power within distribution systems. Utilizing an Enhanced Incremental Conductance (EINC) Maximum Power Point Tracking (MPPT) algorithm, the Photovoltaic (PV) module effectively optimizes power extraction, thereby augmenting the efficiency of the SAPF integration. This system is adept at satisfying the reactive power demands of the load by mitigating harmonics induced by the NLs while concurrently supplying active power harnessed from the PV arrays. The incorporation of the Adaptive Neuro-Fuzzy Inference System (ANFIS) algorithm facilitates the stabilization of the DC link voltage, further contributing to the system’s capability to meet reactive power requirements and elevate grid Power Quality (PQ) through the elimination of harmonics. The proposed strategy effectively reduces harmonics and maintains stable DC link voltage in variable linear and NLs load conditions. This system has been systematically designed, simulated, and experimentally validated, with results across various phases demonstrating its superior performance and enhanced efficiency in improving power quality.

## Introduction

**Fig. 1:**
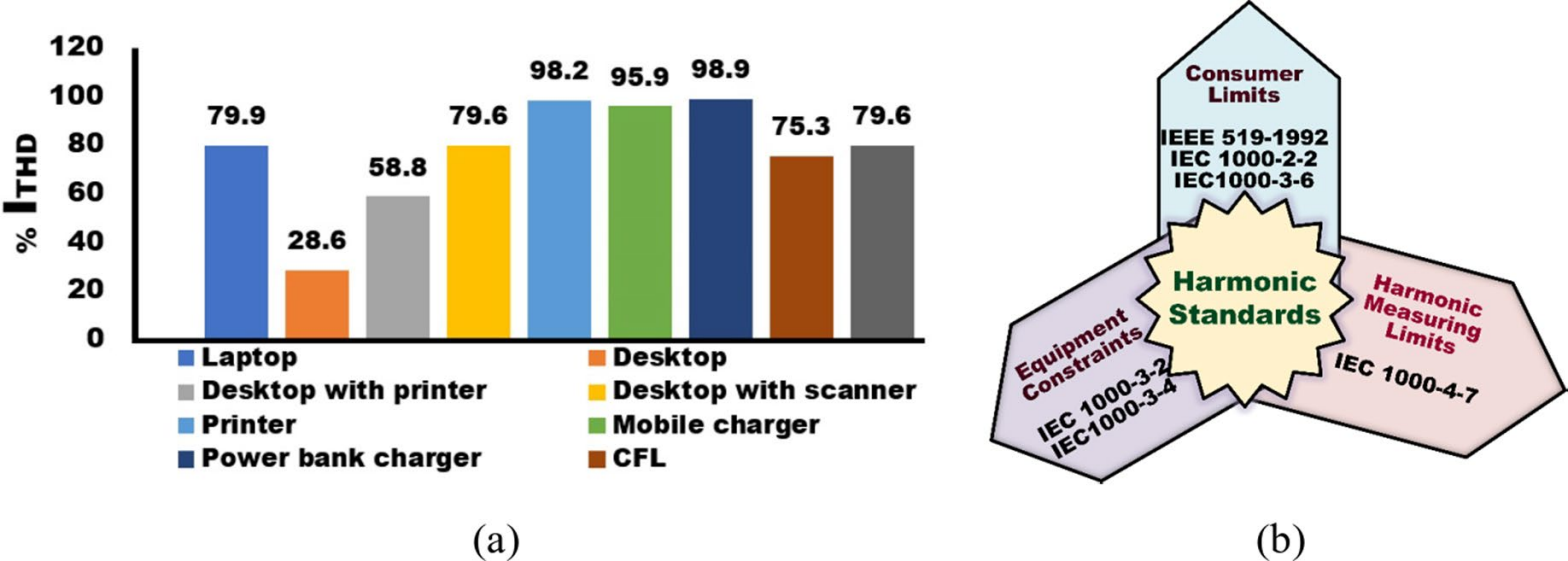
(**a**) Average current total harmonic distortion I_THD_in multiple devices^4^, (**b**) Harmonic Standards.

The power sector plays a crucial role in the economic framework of any nation. The escalation in energy consumption can be directly linked to advancements in living standards and demographic expansion. Consequently, the pursuit of eco-friendly, cost-efficient, and dependable energy sources has emerged as a critical goal for numerous nations globally. The adoption of renewable energy technologies is gaining momentum across different sectors, driven by the finite nature of conventional energy resources and the environmental concerns associated with carbon emissions and the imperative for sustainability^1,2^. Distributed Generation (DG) resources are increasingly recognized as integral to the advancement of modern power systems, given their capacity for efficient energy utilization. However, the synchronization of DG resources within microgrids presents a sophisticated challenge. As industrial growth accelerates and energy consumption escalates, the issue of power quality has emerged as a significant concern among industry specialists. Among the various facets of power quality, power system harmonics stand out as a pivotal concern. The referenced studies delve into this subject, offering comprehensive analyses. The presence of substantial nonlinear demand exacerbates the magnitude of power source harmonics. This contamination’s intensity is subject to fluctuations, influenced by alterations in system configuration, load switching activities, and shifts in the operational modes of demand. With the prevalent use of pulsation loads, which inherently contain harmonic components, there arises the generation of extra frequencies termed as inter-harmonic components. Distinctively, these inter-harmonic components are non-integer multiples of the grid frequency, presenting unique challenges to power quality management^3^. The negative impact of inter-harmonic components on the frequency spectrum can have serious consequences for voltage and current waveforms, thereby adversely affecting the overall integrity of the system. Power grids with harmonic pollution experience a range of issues, including harmonic voltage presence, augmented losses and thermal stress in electrical apparatus, torque perturbations, motor oscillations and acoustical emissions, diminished efficacy of precision instruments, resonance phenomena, and interference with electronic systems, alongside diminished longevity of equipment. Harmonic distortion in electric grids can arise from various factors, including the nonlinear elements in the network and the efficiency of operational equipment. Power converters are a significant source of harmonic generation in electrical networks. Figure 1 (a) displays stochastic measurements using different conventional scenarios of NLs. The IEEE and IEC have developed standards to regulate harmonics in electrical power systems with the goals of ensuring consistency, efficiency, and equipment safety, as shown in Fig. 1 (b) depicting harmonic standards.

Historically, the reduction of harmonics was addressed through the application of passive LC filters, while capacitor banks were predominantly utilized for the compensation of reactive power. Despite their effectiveness, passive filters are beset by several drawbacks, such as their static nature of compensation, the risk of resonance with other elements within the power system, substantial spatial requirements, susceptibility to degradation over time, and the inability to correct load imbalances. Consequently, the research focus has pivoted towards developing of dynamic solutions for harmonic and reactive power compensation. This shift is largely driven by the increased prevalence of power electronic loads and other types of reactive loads, leading to a heightened interest in technologies such as SAPF^5^. For the SAPF to operate optimally, accurately determining the harmonic and reactive current components present in NL loads is essential. Various harmonic extraction techniques have been documented in academic literature to achieve this compensation task, including the PI controller-based technique, instantaneous real and reactive power theory (PQ)^6,7^, the Deadbeat Control Strategy^8^, Synchronous Reference Frame theory (SRF)^9^, includes the instantaneous power theory^10^, and the Discrete Fourier Transform (DFT) approach. Contemporary scholarly articles on PWM converters have documented numerous control algorithms, with their foundational principles varying. These control systems aim to attain an elevated power factor and generate sinusoidal input current waveforms. The voltage-oriented control method represents the predominant approach for indirect power regulation, emphasizing the alignment of the current vector in correspondence to the voltage vector. The realization of unity power factor operation depends on aligning the line current vector with the phase voltage vector of the power supply line. The Direct Power Control (DPC) approach is an innovative control methodology that uses immediate control loops for active and reactive power. It avoids internal current loops and modulator blocks, relying on instantaneous discrepancies between the estimated and the targeted values of power, the voltage vector of the power source, or the virtual-flux vector^11,12^. However, it has drawbacks like high sampling frequency requirements and switching frequency variability. Integrating a predictive control strategy with DPC can mitigate these challenges, enabling superior performance in DPC of a 3φ PWM converter^13,14^.

In high-power industrial applications, both AC and DC drives are commonly used, but they tend to produce unwanted harmonics. Due to the limitations of the switches used, two-level SAPFs are not suitable for mitigating the harmonics created by high-power nonlinear loads. A significant challenge in integrating solar panel power into the grid is reducing THD, which is crucial when delivering electricity to the grid using solar panels. The SAPF is an effective method for reactive power balancing and harmonic mitigation, implemented with Conventionally two-level inverters. This approach is often used for medium power range nonlinear loads, as the high switching frequency of the two-level inverter may pose challenges in handling the power of increased nonlinear loads. Static switches in inverters exhibit significant switch currents, requiring multiple inverter switches to reduce switched currents at a particular power level, increasing switch voltage stress^15^. To mitigate this constraint, a feasible solution involves integrating the solar system with the electrical grid through a multilevel inverter. This approach presents numerous benefits, such as diminished harmonic distortion, decreased switching losses, and enhanced electromagnetic compatibility^16–18^.

This paper presents integrating a PV system with a 3 L NPC inverter of SAPF into the current electrical grid infrastructure to address the concerns raised. The fundamental goal of incorporating this system is to improve power quality and optimize power utilization through the PV array. The collaborative integration of PV systems is expected to smooth the power demand curve, meet peak power demands, and enhance power usage efficiency without overburdening the electrical grid infrastructure. The adoption of this integrated approach is anticipated to ensure reliable mitigation of harmonic distortion and stabilization of the power demand curve. Additionally, this system offers added benefits such as grid current balancing, reactive power compensation, and power factor correction. Considering the importance of clean energy generation, solar energy emerges as an ideal source. However, environmental conditions significantly influence the power output produced by PV panel, leading to variations. To address this, EINC based MPPT algorithms are used to optimize power output, ensuring maximum power extraction from the PV module under fluctuating sunlight levels and ambient temperatures. By effectively switching PV based SAPF inverters, this system can inject PV active power into the PCC while also reducing or offsetting the harmonics produced by NLs. This approach has the potential to minimize costs associated with constructing separate active power filters. A comparison of the environmental and economic aspects of solar photovoltaic renewable energy in relation to conventional fossil fuel-based energy solutions is presented in Table 1. Furthermore, in circumstances where PV power generation is minimal due to shading or overcast conditions, this system can fulfill the role of an active power filter. Consequently, this integrated system proves to be more efficient and cost-effective than standalone PV or standalone APF systems^19–22^.

It is crucial to regulate the capacitor voltage on the inverter’s DC side for the proper functioning of an active power filter. Traditionally, PI controllers have been utilised to manage the voltage on the inverter’s DC side. However, employing PI controllers requires an accurate model, which is difficult to obtain for a system with significant nonlinearity. As a result, various intelligent controllers that do not require precise models have been explored in the literature. These controllers include Particle Swarm Optimization (PSO)-based controllers^23^, Genetic Algorithms (GA)^24^, Fuzzy Logic Control^24,25^, Artificial Neural Network based controls^5,26^, and others. Recent advancements have led to the adoption of the ANFIS approach across a myriad of nonlinear systems to bolster their performance. The PV-SAPF expert system, as introduced, demonstrates proficient handling of unexpected DC link voltage fluctuations ensuing from load variations. Consequently, the adopted strategy necessitates a level of flexibility and responsiveness to the prevailing variables. Fuzzy systems are recognized for their superior capability in managing imprecision and ambiguity, surpassing neural networks in this regard. However, neural networks excel in learning and adapting to novel scenarios. Owing to these considerations, the ANFIS methodology, which amalgamates neural networks and fuzzy logic, has been employed in this study. This approach leverages the strengths of both systems, offering a robust solution.

**Table 1:** Comparison of environmental and economic aspects: solar photovoltaic renewable energy contrasted with conventional fossil fuel-based energy solutions.

Aspect	Solar PV (Renewable Energy)	Conventional Solutions
Greenhouse Gas Emissions	Significantly reduces greenhouse gas emissions, aiding climate change mitigation	Typically results in higher emissions due to dependence on fossil fuels.
Cost Trend	Costs have decreased by approximately 80% over the past decade due to technological advancements.	Costs tend to fluctuate and are often elevated, influenced by fossil fuel market dynamics.
Long-term Savings	Offers substantial long-term savings on electricity bills, complemented by potential government incentives.	Provides limited financial savings, with higher cumulative costs over time
Environmental Impact	Offers substantial long-term savings on electricity bills, complemented by potential government incentives.	Associated with greater environmental degradation from pollution and resource extraction.

**Fig. 2:**
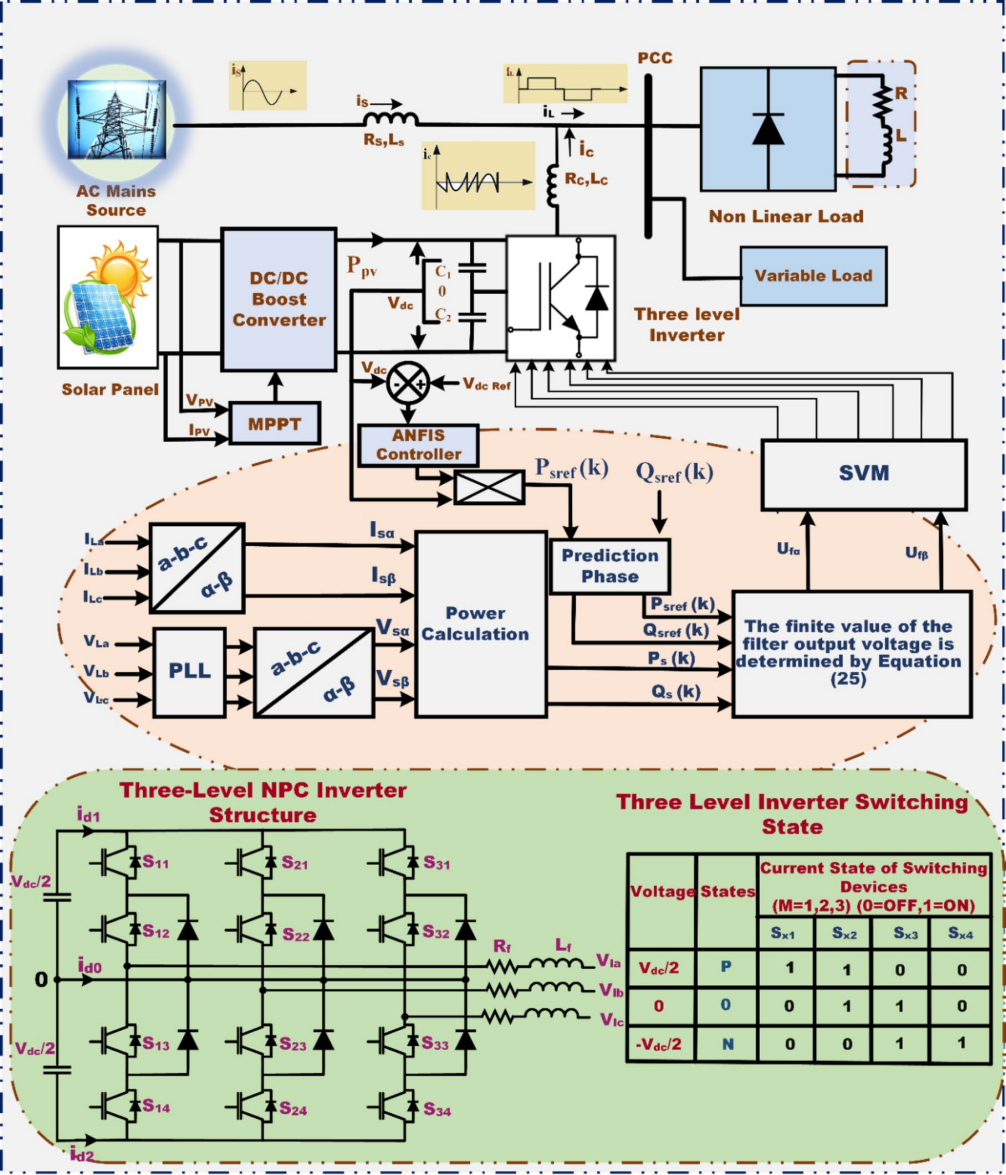
Power circuit diagram of an integrated 3 L NPC inverter-based PV system with SAPF.

The following is a summary of the most significant contributions from the current research:

- The proposes an EINC-based PV interconnection through a three-levels NPC voltage source inverter SAPF to supply active power from the PV system as well as minimise current harmonics while delivering an opposing harmonic current at the Point of common coupling (PCC).
- Secondly, an ANFIS control that relies on novel predictive direct power control strategy will maintain constant DC-link capacitor voltage and optimise PV-SAPF performances in both linear and non-linear load scenarios.
- Thirdly, the proposed system is evaluated with MATLAB/Simulink system performance. The ANFIS controller in SAPF and the conventional PI controller in SAPF are both used to analyse the system performance and the SVM is employed for generating the gating pulses.
- Finally, the performance of a prototype PV-SAPF.is tested and developed in the laboratory. It is proven that the harmonics compensation comes close to meeting IEEE 519 requirements.

## Solar photovoltaic power generation system with DC-DC converter

The proposed photovoltaic system integrated with an NPC-based inverter SAPF system is depicted in Fig. 2. A solar PV system utilises solar energy to produce electricity by employing one or more linked PV modules or panels. The primary system usually consists of solar panels, along with the necessary mechanical and electrical connections, to generate electricity. PV production is often influenced by solar irradiance, which is affected by weather conditions. The solar photovoltaic system uses an incremental conductance MPPT controller to maximize the power output of the photovoltaic array. This algorithm optimizes power extraction from the solar photovoltaic array so that the PV panel yields maximum power. The solar PV system is coupled with a SAPF via a DC-DC boost converter to support a DC-link capacitor voltage for compensation^27–30^. The solar integrated shunt APF effectively addresses issues related to harmonic distortion and reactive power imbalance generated by non-linear loads. It achieves this by actively infusing compensating currents into the system to correct these issues and enhance the overall electricity quality. Integrating a SAPF into a PV system enhances the efficient utilization of solar energy while concurrently mitigating power quality concerns.

### Modelling of solar PV boost converter

**Fig. 3:**
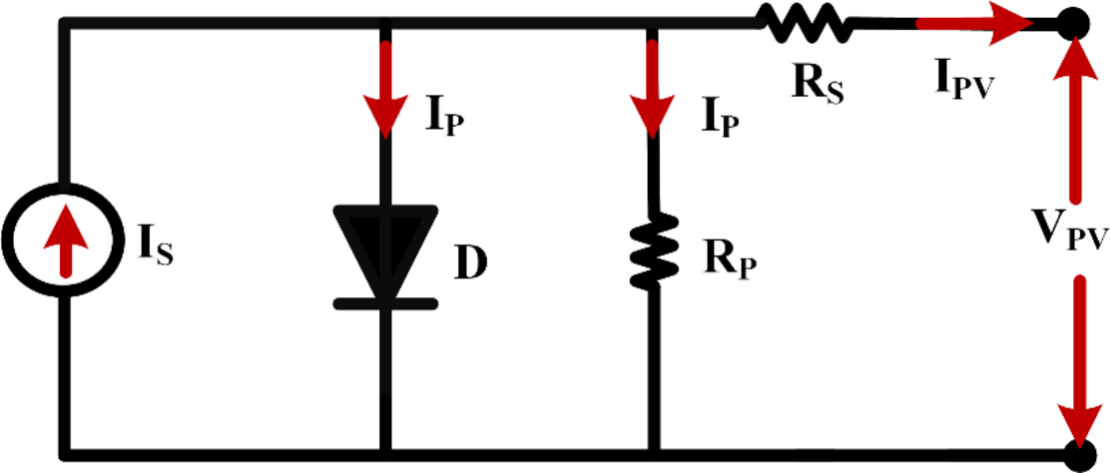
Equivalent circuit of solar PV.

In the study of solar photovoltaic boost converters, it is crucial to take into account factors such as temperature, irradiance levels, and the configuration of strings arranged in both series and parallel formations. These factors significantly influence the current and voltage properties of the PV array. Consequently, the selection of an appropriate solar panel is of paramount importance. For our purposes, the Trina Solar TSM-200 DC/DA01A panel has been chosen. An equivalent model of the solar PV system is depicted in Fig. 3.

The electrical resistances of cells connected in parallel (denoted as n_p_) and those arranged in series (represented as n_s_), respectively, whereas the diode is denoted by the character D. Diode current is determined based on an ideal PV circuit is

1 $$\:{i}_{d}={I}_{0}\left({e}^{\frac{{V}_{D}}{\gamma\:{V}_{T}}}\:-1\right)$$

In the given equation, the symbol γ represents the ideality factor, I_0_ represents the saturation current, and the thermal voltage $\:{V}_{T}=\left(\frac{\text{K}{\text{T}}_{c}}{q}\right)$ is determined by the charge of the electron q, the cell’s operational temperature (Tc), and Boltzmann’s constant (k).

Output power

2 $$\:{P}_{PV}={V}_{PV}*{I}_{PV}$$

Output Current is

3 $$\:{I}_{PV}={I}_{S}-{I}_{d}-{I}_{p}={I}_{S}-{I}_{0}\left({e}^{\frac{{V}_{D}}{\gamma\:{V}_{T}}}\:-1\right)-{I}_{p}$$

4 $$\:{I}_{PV}={n}_{p}{I}_{S}-{n}_{p}{I}_{0}\left({e}^{\left(\frac{1}{\gamma\:{V}_{T}}\right)\left(\frac{{V}_{PV}}{{n}_{s}}+\frac{{R}_{s}{I}_{pv}}{{n}_{p}}\right)}\:-1\right)-\frac{{n}_{p}}{{R}_{p}}\left(\frac{{V}_{PV}}{{n}_{s}}+\frac{{R}_{s}{I}_{pv}}{{n}_{p}}\right)$$

Short circuit current is

5 $$\:{I}_{s\left(T\right)}={I}_{s\left(TR\right)}\left[\beta\:\left(T-{T}_{R}\right)\right]+1$$

The PV data sheets meticulously enumerate the values for the temperature coefficient, denoted as B, alongside the reference temperature, symbolized as TR. Additionally, they furnish the data for the short circuit current, I_S(TR)_, which correlates to the reference temperature.

6 $$\:{I}_{s\left(G\right)}=\frac{G}{{G}_{n}}$$

In the given context, G_n_ represents the standard irradiance value. The saturation current, denoted as I_0_, is determined when the photovoltaic current I_PV_ equals zero. Consequently, the value of I_0_ at the reference temperature, TR is established by the following expression:

7 $$\:{I}_{s\left(G\right)}=\frac{{I}_{s}}{\left({e}^{\frac{{V}_{D}}{\gamma\:{V}_{T}}}\:-1\right)}$$

### Modelling of DC-DC boost converter

The boost converter incorporates an enhanced incremental and conductance MPPT algorithm to automatically track the MPP of the PV array. When switch S is turned on, the output state becomes isolated as the switch and V_c_ reverse bias the diode D, causing the switch to be in an isolated state. Inductor L alongside switch S serve as the pathways for the higher input current I_PV_. The inductor draws energy from the input V_pv_ during the ON time. The voltage of the inductor can be represented as

8 $$\:{V}_{L}=L\frac{di}{dt}$$

During the period characterized by the switch S being in the off state, it is imperative for the current traversing the inductor to be maintained through the conduit of both the diode D and the load. The diode D facilitates the establishment of a serial linkage between the inductor L and the voltage source, ensuring a continuous flow of current. Should there be a diminution in the current’s magnitude, the corresponding electromagnetic field generated by the inductor is subjected to a polarity reversal, highlighting the dynamic interplay between the circuit components and the governing electromagnetic principles. The design of the inductor for a boost converter is predicated upon the utilization of the following mathematical formula:

9 $$\:{L}_{b}=\frac{{V}_{in}D}{{f}_{sw}\nabla\:{i}_{L}}$$

The input voltage V_pv_ is V_in_, the switching frequency is f_sw_, the duty cycle is D=$\:\frac{{\varvec{V}}_{\varvec{i}\varvec{n}}}{\left({\varvec{V}}_{\varvec{o}\varvec{u}\varvec{t}}-1\right)}$, and the present oscillation observed within the inductor of the boost converter is denoted as Δi_L_.

### Maximum power point tracking

**Fig. 4:**
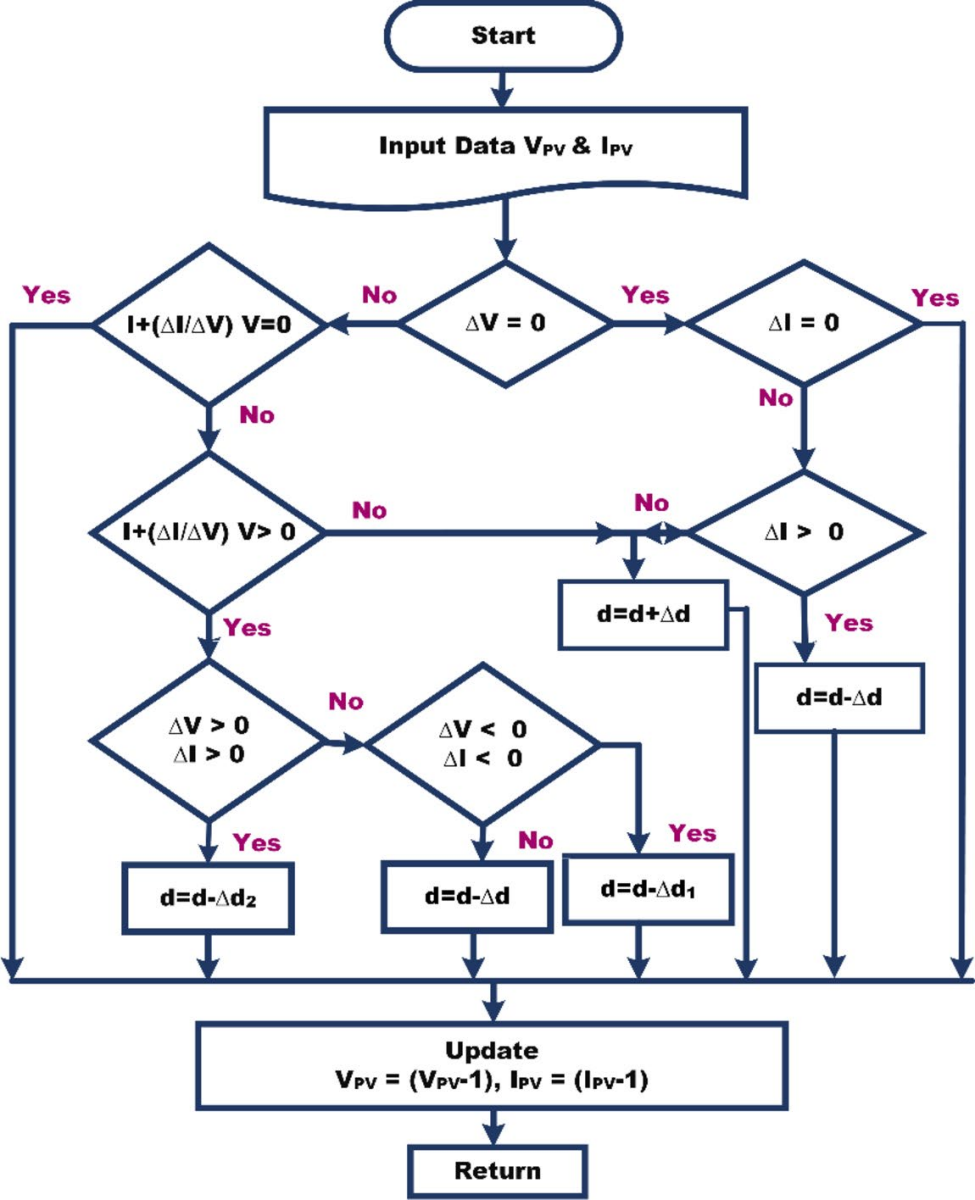
EINC MPPT technique.

The MPPT controller takes into account environmental factors, including solar irradiance and ambient temperature, alongside the distinct parameters of the PV array, such as open-circuit voltage and short-circuit current, in addition to the voltage of the DC link. This comprehensive evaluation enables the MPPT controller to optimize the operation of the boost converter with remarkable efficiency. This controller is responsible for supervising and regulating the operation of the boost converter. Conventional incremental and conductance MPPT algorithms become less effective when the operating point fluctuates around the MPP and there are rapid changes in irradiance conditions. In response to these challenges, an EINC-based MPPT technique has been implemented^31^. In Table 2, a comprehensive comparison of various MPPT techniques has been undertaken. This comparison evaluates several key features, including control strategy, implementation complexity, circuit configuration, cost, as well as pros and cons. The focus is on assessing the efficacy of different MPPT techniques under conditions of normal irradiance. Figure 4 illustrates the detailed EINC method that is executed.

**Table 2:** Comparison of MPPT techniques under standard irradiance conditions^31–33^.

Comparison parameter	MPPT Algorithm					
	Curve Fitting	*P*&O	INC	ANN	FLC	EINC
PV Array Dependent	Yes	No	No	Yes	Yes	No
Sensed parameters	V or I	V, I	V, I	V, I, G	V, I or G, T	V, I
Analog or Digital Control	Digital	Both	Digital	Digital	Digital	Digital
Convergence speed	Slow	Varies	Varies	Fast	Fast	moderate
Complexity	Simple	Simple	medium	complex	complex	medium
Parameter Tuning	Yes	No	No	Yes	Yes	No
Cost	Inexpensive	Expensive	Expensive	Expensive	Expensive	Expensive
Pros	Use simple logic	Good tracking capability	Low oscillation near MPP	Good response and less oscillation near MPP	Good response and little oscillation near MPP	Good tracking capability
Cons	Continuous power loss	Continuous oscillation and tracking speed	Complexity in implementation	Parameter tuning	Complexity in implementation and selection of proper range of operation	Complexity in implementation

## Proposed control of PV integrated SAPF

The inverter architecture incorporates a total of twelve active switches alongside six clamping diodes. On the DC side, it features a pair of identical capacitors that establish the DC-link. The midpoint between these capacitors is denoted as the neutral-point, playing a critical role in the system’s functionality. Each operational switch is paired with a diode in antiparallel configuration to facilitate bidirectional current flow. In practical implementations, it is feasible to utilize either an Insulated Gate Bipolar Transistor (IGBT) or a similarly purposed switching device, underscoring their versatility in controlling power distribution efficiently. The switching state variable, denoted as Sx, is employed to represent the switching state of phase x. It can take on three potential values, represented by the symbols +, 0, and −. These values correspond to the switching configurations that yield Vdc/2, 0, and -Vdc/2, at the phase output of the inverter, respectively. To facilitate the representation of the 3φ output voltages as space vectors within an orthogonal plane, the vector model of the 3 L NPC converter is employed. This 3 L inverter produces twenty-seven switching states, leading to the generation of nineteen distinct voltage vectors.

### **Principle of proposed PDPC-SVM strategy for SAPF**

The conventional Direct Power Control (DPC) method presents a notable limitation concerning the uncontrollable switching frequency, which necessitates a high sampling rate for the accurate and efficient regulation of active and reactive powers^34^.To address the significant drawback associated with DPC’s reliance on a predetermined switching table, it is crucial to develop a novel configuration that establishes specific criteria for the quality of regulated powers. This approach is known as Predictive Direct Power Control (PDPC)^12^. Furthermore, the proposed methodology has been applied to the regulation of SAPF, ensuring improved energy quality, as illustrated in Fig. 2. Predictive control systems have increasingly gained popularity during the past thirty years. The PDPC-SVM technique relies on predicting real-time active and reactive power metrics utilising a sophisticated predictive model. This technique plays a pivotal role in the management of the voltage source inverter, wherein it leverages an average voltage vector throughout every sampling interval. This average voltage vector is then translated into a series of adjacent control vectors for the attainment of the requisite active and reactive powers^35^. The outer control loop of the DC link capacitor voltage enables the precise determination of the active power’s current magnitude, while the reactive power is rigorously controlled to closely approach zero, thus aiding in the improvement of power factor correction.

A predictive approach primarily relies on forecasting the control variables that need to be implemented within a certain sample period to achieve the target level of performance. In order to accomplish that objective, several quantities need to be determined. In the advanced predictive model being proposed, the variable parameters subject to regulation encompass both active and reactive power components. The formulation of active and reactive power within a stationary reference frame, denoted as (α, β), for a balanced 3φ system, is articulated as follows^36^:

10 $$\:\left[\begin{array}{c}{P}_{s}\\\:{Q}_{s}\end{array}\right]=\left[\begin{array}{cc}{V}_{s\alpha\:}&\:{V}_{s\beta\:}\\\:{V}_{s\beta\:}&\:{-V}_{s\alpha\:}\end{array}\right]\left[\begin{array}{c}{i}_{s\alpha\:}\\\:{i}_{s\beta\:}\end{array}\right]$$

The fluctuation of instantaneous active and reactive powers may be determined by taking the time derivative in the following manner:

11 $$\:\frac{d}{dt}\left[\begin{array}{c}{P}_{s}\\\:{Q}_{s}\end{array}\right]=\frac{d}{dt}\left[\begin{array}{cc}{V}_{s\alpha\:}&\:{V}_{s\beta\:}\\\:{V}_{s\beta\:}&\:{-V}_{s\alpha\:}\end{array}\right]\left[\begin{array}{c}{i}_{s\alpha\:}\\\:{i}_{s\beta\:}\end{array}\right]\:+\:\left[\begin{array}{cc}{V}_{s\alpha\:}&\:{V}_{s\beta\:}\\\:{V}_{s\beta\:}&\:{-V}_{s\alpha\:}\end{array}\right]\frac{d}{dt}\left[\begin{array}{c}{i}_{s\alpha\:}\\\:{i}_{s\beta\:}\end{array}\right]$$

The discretization of Eq. (11) yields the following results when the sampling period is much smaller than the fundamental period:

12 $$\:\left[\begin{array}{c}{\text{P}}_{\text{s}}(\text{k}+1)\:-{\:\text{P}}_{\text{s}}\left(\text{k}\right)\\\:{\text{Q}}_{\text{s}}(\text{k}+1)\:-{\:\text{Q}}_{\text{s}}\left(\text{k}\right)\end{array}\right]=\:\left[\begin{array}{cc}{V}_{s\alpha\:}&\:{V}_{s\beta\:}\\\:{V}_{s\beta\:}&\:{-V}_{s\alpha\:}\end{array}\right]\:\left[\begin{array}{cc}{i}_{s\alpha\:}(k+1)&\:{i}_{s\alpha\:}\left(k\right)\\\:{i}_{s\beta\:}(k+1)&\:{-\text{i}}_{s\beta\:}\left(k\right)\end{array}\right]$$

In the SAPF system, the load incurs a non-sinusoidal current, predominantly comprising the current originating from the source and the current engendered by the filter. The source is tasked with furnishing the fundamental current, whereas the filter undertakes the responsibility of providing the harmonic currents, as detailed subsequently.

13 $$\:{\text{i}}_{\text{l}\text{x}}={\text{i}}_{\text{s}\text{x}}+{\text{i}}_{\text{f}\text{x}}$$

14 $$\:{\text{i}}_{\text{l}\text{x}}={\text{i}}_{\text{F}\text{u}\text{n}\text{d}\text{a}\text{m}\text{e}\text{n}\text{t}\text{a}\text{l}\:\text{x}}+{\text{i}}_{\text{H}\text{a}\text{r}\text{m}\text{o}\text{n}\text{i}\text{c}\:\text{x}}$$

15 $$\:{\text{i}}_{\text{f}\text{x}}-{\text{i}}_{\text{H}\text{a}\text{r}\text{m}\text{o}\text{n}\text{i}\text{c}\:\text{x}}={\text{i}}_{\text{F}\text{u}\text{n}\text{d}\text{a}\text{m}\text{e}\text{n}\text{t}\text{a}\text{l}\:\text{x}}\:-{\text{i}}_{\text{s}\text{x}}$$

Where, x = a, b, c.

The observed fluctuations in the filter current exhibit an identical magnitude relative to the source current, albeit in the inverse direction.

16 $$\:{\text{i}}_{\text{f}\text{x}}-{\text{i}}_{\text{H}\text{a}\text{r}\text{m}\text{o}\text{n}\text{i}\text{c}\:\text{x}}=-\left({-{\text{i}}_{\text{s}\text{x}}-\text{i}}_{\text{F}\text{u}\text{n}\text{d}\text{a}\text{m}\text{e}\text{n}\text{t}\text{a}\text{l}\:\text{x}}\right)\:$$

Then

17 $$\:\varDelta\:{\text{i}}_{\text{f}\text{x}}=-\varDelta\:{\text{i}}_{\text{s}\text{x}}$$

For slight fluctuations in both source and filter currents, it can be posited that Eq. (17) can be characterized as follows

18 $$\:\text{d}{\text{i}}_{\text{f}\text{x}}=-{\text{d}\text{i}}_{\text{s}\text{x}}$$

The final equation provides the determination of both the variations in filter current and the voltage at PCC without requiring direct measurement of the filter current. As a result, it can be reformulated as:

19 $$\:{\text{L}}_{\text{f}}\frac{{\text{d}\text{i}}_{\text{s}\text{x}}}{\text{d}\text{t}}={\text{V}}_{\text{s}\text{x}}-{\text{U}}_{\text{f}\text{x}}$$

The following formula can be used to express the variations in the source current within the α-β reference frame.

20 $$\:\left[\begin{array}{c}{\text{i}}_{\text{s}{\upalpha\:}}(\text{k}+1)\:-{\text{i}}_{\text{s}{\upalpha\:}}\left(\text{k}\right)\\\:{\text{i}}_{\text{s}{\upbeta\:}}(\text{k}+1)\:-{\text{i}}_{\text{s}{\upbeta\:}}\left(\text{k}\right)\end{array}\right]=\frac{{T}_{s}}{{L}_{f}}\left(\left[\begin{array}{c}{V}_{s\alpha\:}\left(k\right)\\\:{V}_{s\beta\:}\left(k\right)\end{array}\right]-\left[\begin{array}{c}{U}_{f\alpha\:}\left(k\right)\\\:{U}_{f\beta\:}\left(k\right)\end{array}\right]\right)$$

By utilizing the predictive model, which is formulated through the substitution of the preceding equation into Eq. (12), it becomes possible to accurately predict the values of both active and reactive powers for SAPF.

21 $$\:\left[\begin{array}{c}{\text{P}}_{\text{s}}(\text{k}+1)\:-{\:\text{P}}_{\text{s}}\left(\text{k}\right)\\\:{\text{Q}}_{\text{s}}(\text{k}+1)\:-{\:\text{Q}}_{\text{s}}\left(\text{k}\right)\end{array}\right]=\frac{{T}_{s}}{{L}_{f}}\:\left[\begin{array}{cc}{V}_{s\alpha\:}\left(k\right)&\:{V}_{s\beta\:}\left(k\right)\\\:{V}_{s\beta\:}\left(k\right)&\:{-V}_{s\alpha\:}\left(k\right)\end{array}\right]\left(\left[\begin{array}{c}{V}_{s\alpha\:}\left(k\right)\\\:{V}_{s\beta\:}\left(k\right)\end{array}\right]-\left[\begin{array}{c}{U}_{f\alpha\:}\left(k\right)\\\:{U}_{f\beta\:}\left(k\right)\end{array}\right]\right)$$

The primary characteristics that significantly impact the prediction stage are the sample time, filter, and source inductance. The predictive algorithm aims to reduce the discrepancy during the reference and actual observed values of both active and reactive power, striving to achieve a difference as close to zero as possible.

22 $$\:\left\{\begin{array}{c}{\epsilon\:}_{p}\left(k\right)={P}_{sref}\left(k\right)-{P}_{s}\left(k\right)\approx\:0\\\:{\epsilon\:}_{q}\left(k\right)={Q}_{sref}\left(k\right)-{Q}_{s}\left(k\right)\approx\:0\end{array}\right.$$

For achieving the desired power values, it is important to ensure that the following equation is satisfied to achieve an optimal convergence.

23 $$\:\left\{\begin{array}{c}{\text{P}}_{\text{s}}(k+1)-{P}_{sref}(k+1)=0\\\:{\text{Q}}_{\text{s}}(k+1)\:-{\:Q}_{sref}(k+1)=0\end{array}\right.$$

Then

24 $$\:\left\{\begin{array}{c}{\text{P}}_{\text{s}}(k+1)={P}_{sref}(k+1)\\\:{\text{Q}}_{\text{s}}(k+1)={Q}_{sref}(k+1)\end{array}\right.$$

Applying Eq. (24) to the Eq. (21):

25 $$\:\left[\begin{array}{c}{P}_{sref}(k+1)\:-{\:P}_{s}\left(k\right)\\\:{\:Q}_{sref}(k+1)-{Q}_{s}\left(k\right)\end{array}\right]=\frac{{T}_{s}}{{L}_{f}}\:\left[\begin{array}{cc}{V}_{s\alpha\:}\left(k\right)&\:{V}_{s\beta\:}\left(k\right)\\\:{V}_{s\beta\:}\left(k\right)&\:{-V}_{s\alpha\:}\left(k\right)\end{array}\right]\left(\left[\begin{array}{c}{V}_{s\alpha\:}\left(k\right)\\\:{V}_{s\beta\:}\left(k\right)\end{array}\right]-\left[\begin{array}{c}{U}_{f\alpha\:}\left(k\right)\\\:{U}_{f\beta\:}\left(k\right)\end{array}\right]\right)$$

**Fig. 5:**
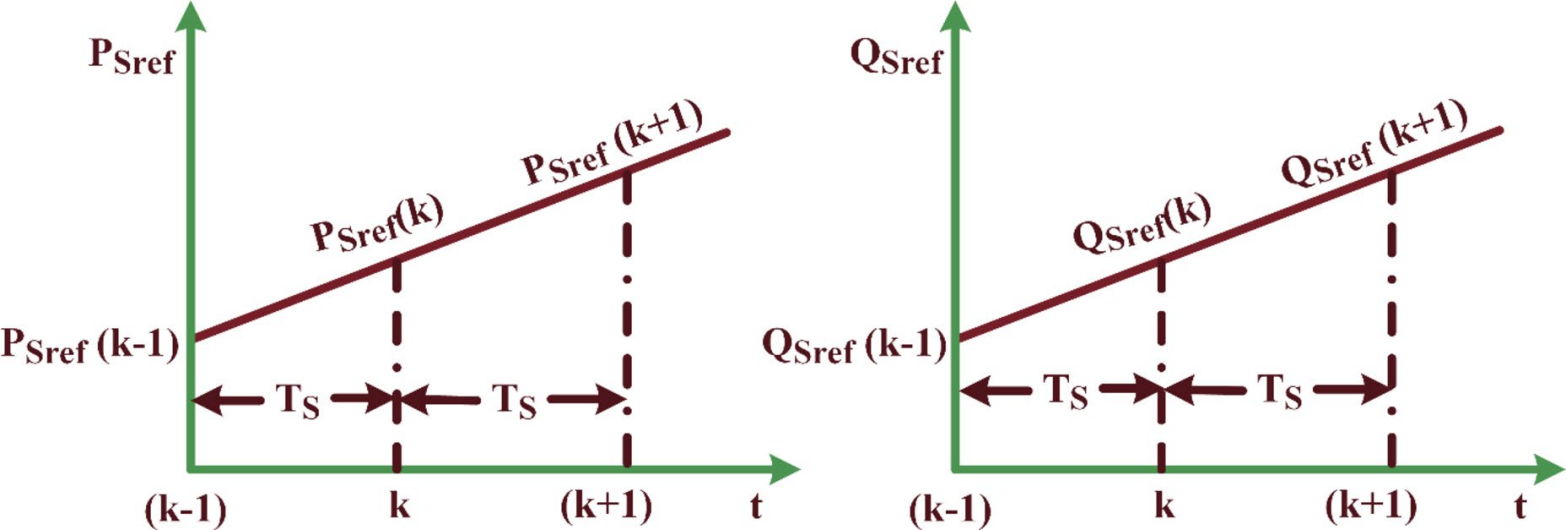
Estimation of predictive value in reference power.

It is important to acknowledge that the reference value of active power undergoes linear variation during two consecutive sampling intervals, as delineated in Fig. 5. This This observation assumes that the voltage regulation loop’s inaccuracy does not fluctuate over the successive sampling periods. In pursuit of attaining a power factor of unity, it is customary to assign a fixed value to the reference of reactive power, generally established as zero. Consequently, this protocol enables the accurate prediction of predefined values for both active and reactive power for the ensuing sampling interval, employing a designated methodology.

26 $$\:\left[\begin{array}{c}{P}_{sref}(k+1)\:)\\\:{\:Q}_{sref}(k+1)\end{array}\right]=\:\left[\begin{array}{c}2{P}_{sref}\left(k\right)-{P}_{sref}(k-1)\\\:{Q}_{sref}\left(k\right)\end{array}\right]$$

The formulation to derive the average control vector, intended for application across the sampling interval ([kTs, (k + 1) Ts]), can be achieved by employing Eqs. (25) and (26).

27 $$\:\left[\begin{array}{c}{U}_{f\alpha\:}\left(k\right)\\\:{U}_{f\beta\:}\left(k\right)\end{array}\right]=\left[\begin{array}{c}{V}_{s\alpha\:}\left(k\right)\\\:{V}_{s\beta\:}\left(k\right)\end{array}\right]-\frac{{L}_{f}}{{T}_{s}({V}_{s\alpha\:}^{2}+{V}_{s\beta\:}^{2})}\left[\begin{array}{cc}{V}_{s\alpha\:}\left(k\right)&\:{V}_{s\beta\:}\left(k\right)\\\:{V}_{s\beta\:}\left(k\right)&\:{-V}_{s\alpha\:}\left(k\right)\end{array}\right]\:\left[\begin{array}{c}2{P}_{sref}\left(k\right)-{P}_{sref}(k-1)-{P}_{s}\left(k\right)\\\:{Q}_{sref}\left(k\right)-{Q}_{s}\left(k\right)\end{array}\right]$$

**Fig. 6:**
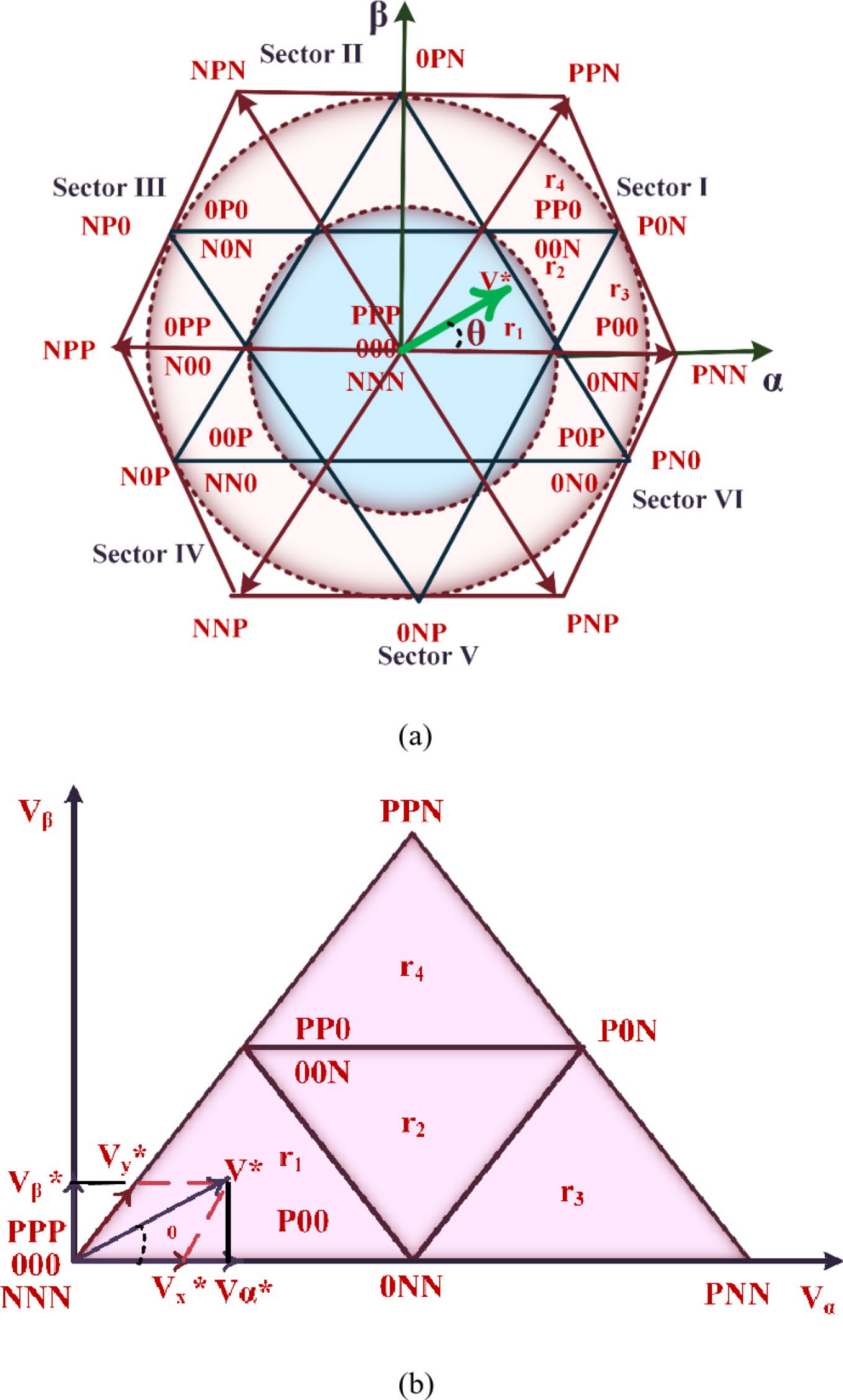
(**a**) Three-level NPC inverter space-vector diagram. (**b**) Analysis of Sector 1 for Estimation of Voltage Vector Component.

The primary aim of the PDPC technique is to accurately compute the reference voltage values. Subsequently, these calculated values serve as critical inputs for the Space Vector Modulation (SVM) methodology. In comparison with the traditional Pulse Width Modulation technique, the SVM approach significantly enhances efficiency by mitigating current fluctuations. This study employed SVM technique to regulate the pulse generation of the three-level SAPF. The primary aim of the SVM method is to accurately determine a predefined sequence of inverter states. This intricate process encompasses multiple stages, such as the identification of the specific sector and region, calculation of switching times, and the determination of appropriate switching sequences^37–40^. Additionally, the procedure involves the establishment of the reference voltage vector, followed by the generation of Pulse Width Modulation signals. The 3 L inverter structure comprises a total of twenty-seven vectors, of which twenty-four are characterized as active. These active vectors are further classified into three distinct categories based on their magnitude: twelve are identified as short vectors, six as medium vectors, and six as long vectors. Additionally, the inverter architecture incorporates three zero vectors, which play a crucial role in its operational dynamics. In Fig. 6 (a), all lines converge at the central point of a hexagon. this intricate configuration forms a hexagonal architecture, strategically segmented into six sectors. Every sector is subdivided into four sections, optimizing the deployment of synergetic control mechanisms within the αβ reference frames. The deployment of reference voltage vectors is meticulously articulated in the αβ reference frames, defined by precise magnitude and angular specifications.

28 $$\:\left|{V}^{*}\right|=\sqrt{{V}_{f\alpha\:}^{2}+{V}_{f\beta\:}^{2}};\theta\:=atan\left(\frac{{V}_{f\alpha\:}}{{V}_{f\alpha\:}}\right)$$

29 $$\:{S}_{k}=ceil\left(\frac{\theta\:}{\pi\:/3}\right)\in\{\text{1,2},\text{3,4},\text{5,6}\}$$

The “ceil” function rounds a real value up to the closest integer. The estimated voltage vector components$\:{V}_{x}^{*}$_,_ $\:{V}_{y}^{*}$, and $\:{V}^{*}$ are ascertained through the analysis of sector 1 depicted in Fig. 6(b).

30 $$\:\left\{\begin{array}{c}{V}_{x}^{*}=\left|{V}^{*}\right|\left(cos\right(\theta\:)-\frac{1}{\sqrt{3}}sin(\theta\:\left)\right)\\\:{V}_{y}^{*}=\frac{\sqrt{3}}{2}\left|{V}^{*}\right|sin\left(\theta\:\right)\end{array}\right.$$

31 $$\:\:{\text{I}\text{f}\:{V}_{x}^{*}<{0.5}^{*}\sqrt{\frac{2}{3}}{V}_{dc\:};V}_{y}^{*}<{0.5}^{*}\sqrt{\frac{2}{3}}{V}_{dc\:};\:{(V}_{x}^{*}+{V}_{y}^{*})<{0.5}^{*}\sqrt{\frac{2}{3}}{V}_{dc\:};{r}_{1}$$

32 $$\:\text{I}\text{f}\:{V}_{x}^{*}>{0.5}^{*}\sqrt{\frac{2}{3}}{V}_{dc\:};\:{r}_{2}$$

33 $$\:\text{I}\text{f}\:{V}_{x}^{*}<{0.5}^{*}\sqrt{\frac{2}{3}}{V}_{dc\:};\:{V}_{y}^{*}<{0.5}^{*}\sqrt{\frac{2}{3}}{V}_{dc\:};\:{(V}_{x}^{*}+{V}_{y}^{*})>{0.5}^{*}\sqrt{\frac{2}{3}}{V}_{dc\:};{r}_{3}$$

34 $$\:\text{I}\text{f}\:{V}_{y}^{*}>{0.5}^{*}\sqrt{\frac{2}{3}}{V}_{dc\:};\:{r}_{4}$$

The methodology employed for computing switching times maintains uniformity across various industrial sectors, despite the occurrence of variations in specific values within identical sectors, contingent upon the geographical region. It is imperative to contemplate both the total cycle duration and the durations of adjacent vectors for the accurate determination of switching times.

35 $$\:\left\{\begin{array}{c}{TV}^{*}={T}_{x}{V}_{1}+{T}_{y}{V}_{2}+{T}_{z}{V}_{0}\\\:T={T}_{x}+{T}_{y}+{T}_{z}\\\:{V}^{*}=\left|{V}^{*}\right|{e}^{j\theta\:},\theta\:=\angle\:{V}^{*}\end{array}\right.$$

### Control of DC bus voltage

There are two primary functions that the DC side capacitor performs: first, it sustains a direct current voltage characterized by minimal ripple under steady-state conditions; second, it acts as an energy storage mechanism that compensates for the real power discrepancy across the load and the source amidst transitory events. These critical roles underscore the indispensability of the DC side capacitor in ensuring system stability and efficiency. To ensure a stable operational state, the power provisioned by the source must be congruent with the actual power requisites of the load, supplemented by an additional quantum of power designed to offset the losses incurred by the active filter. Consequently, this arrangement facilitates the stabilization of the DC capacitor voltage at a predetermined reference value. Nonetheless, alterations in the load conditions precipitate a disruption in the equilibrium of real power between the main power source and the load. The resultant disparity in real power necessitates compensation via the DC capacitor, thereby deviating the DC capacitor voltage from its designated reference voltage. To uphold optimal functionality of the active filter under such circumstances, it is imperative that the peak magnitude of the reference current is modulated to effectuate a corresponding adjustment in the real power extracted from the source. The real power thus absorbed or dispensed by the capacitor serves to counterbalance the real power expenditure of the load. Upon the restoration of the DC-link voltage to its reference value, it is inferred that the real power emanating from the source has been harmonized with the consumption metrics of the load. The regulation of the direct current voltage within a SAPF constitutes a critical phase to guarantee optimal system performance. A DC bus control loop is imperative for maintaining voltage stability, thereby ensuring efficient operation in a steady-state condition. This mechanism is predicated on the principle of energy balance stored within the capacitor, which is aligned with the DC link reference voltage (V_dc ref_).

**Fig. 7:**
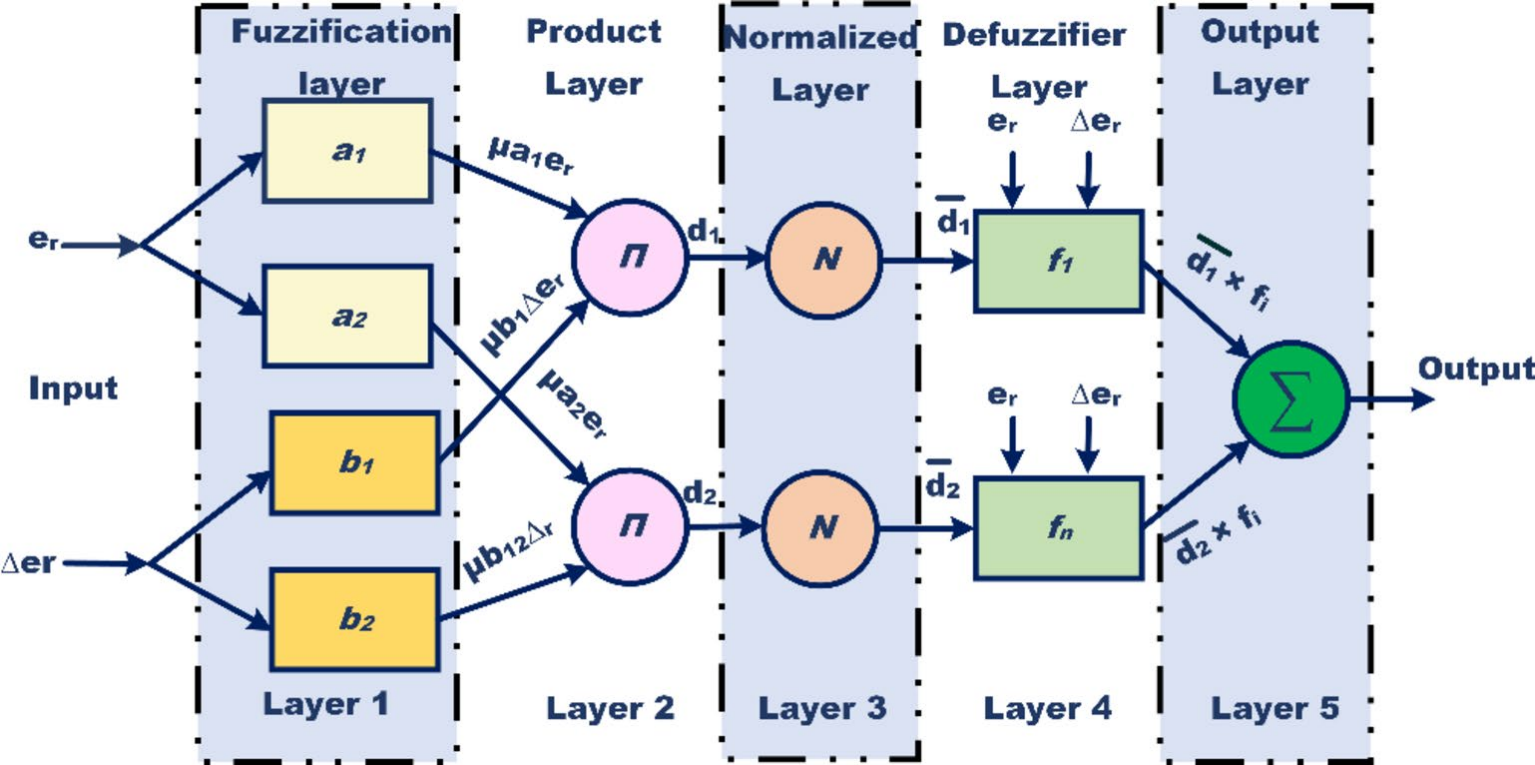
ANFIS controller structure.

**Fig. 8:**
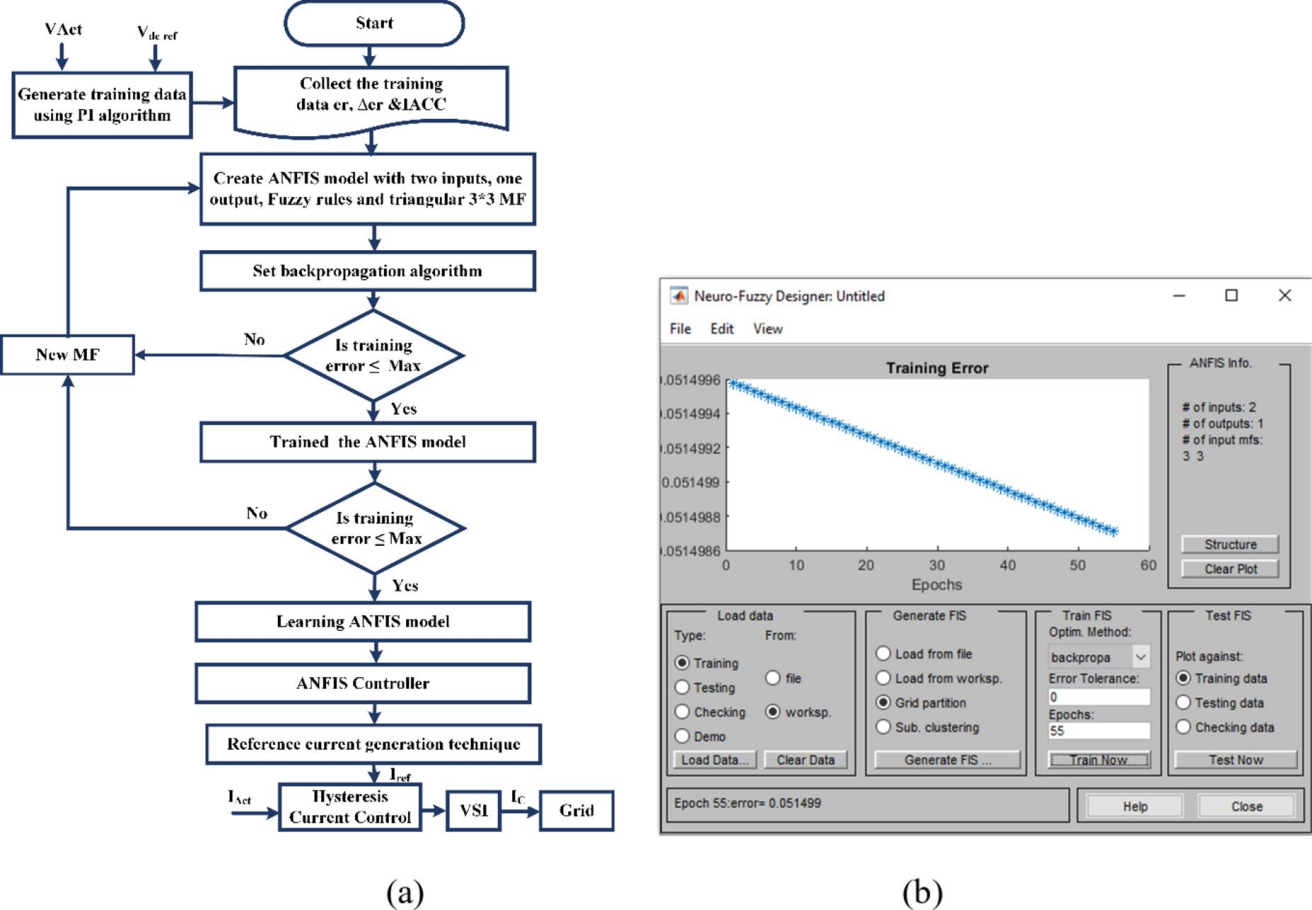
ANFIS Controller: (**a**) Flowchart for tuning algorithm (**b**) Epochs and training errors of ANFIS controller.

The implementation of the ANFIS algorithm for the regulation of dc-link capacitor voltage of SAPF significantly contributes to the enhanced power quality. The training data for the ANFIS controller is initially derived from a proportional-integral (PI) controller, with proportional gain (K_p_) set to 0.16 and integral gain (K_i_) set to 0.42. The PI controller undergoes simulation within a MATLAB environment and is subjected to various load scenarios to generate the requisite training data in conjunction with a SAPF controller. In the subsequent phase, an ANFIS model is constructed that employs membership functions based on Sugeno fuzzy rules, incorporating one output variable and two input variables, namely the error and the change in error. Figure 7 presents the architecture of the five-layer ANFIS algorithm. The ANFIS architecture utilizes a 3 × 3 matrix of triangular membership functions. The learning procedure of the ANFIS model is implemented through a backpropagation algorithm, trained for 55 epochs, optimizing the model to minimize the training error. This iterative training process continues until the deviation between the target data and the ANFIS output is sufficiently reduced. Ultimately, the fuzzified outputs from the ANFIS model are converted into crisp variables, which are then employed by the SAPF controller to effectively decrease THD and enhance PQ. Illustratively, Fig. 8 (a) delineates a comprehensive flowchart that captures the sequential tuning of SAPF for power quality enhancement through the application of the ANFIS algorithm. Figure 8(b) outlines the architecture and projected power output in the context of PV-SAPF control operation amidst ANFIS training, incorporating both epoch and training data errors for a comprehensive understanding and highlighting the estimated Root Mean Square Error of the ANFIS model calculated at 0.051499.

## Simulation results and discussion

In this study, a sophisticated simulation model of the SAPF scheme has been meticulously developed utilizing the MATLAB environment. This comprehensive model includes several critical components: a three-phase AC source, a photovoltaic interfacing system equipped with a 3 L NPC based inverter, coupling inductance, nonlinear loads, and an advanced control unit. To ensure a thorough understanding and replication of the research, all parameters pertinent to the simulation model have been systematically documented in Table 3.

**Table 3:** Design the parameters of the simulation.

Parameter	Value
Source voltage (Phase voltage)	230 V,50 Hz
PV Boost converter	
PV Module	P_PV_=200.9 W; I_sc_=5.62 A; V_oc_=46.2 V; V_m_=38.2 V; I_m_=5.26 V.
Boost Converter	L = 68.5 mH; C_in_= 100 µF; V_pv_=V_in_=305.6 V; V_dc_=750 V
Filter Inductance	5 mH
DC-link Capacitor	2200µF
Nonlinear load	
Resistor and inductor	*R* = 81Ω and L = 12mH
Balanced Linear load	
Resistive load	*R* = 55 Ω and 110 Ω
Inductive load	140mH

Note: V_m_, I_m_ and P_PV_ are maximum power voltage, current and power of PV; Voc and Isc are open-circuit voltage and Short-circuit current.

**Fig. 9:**
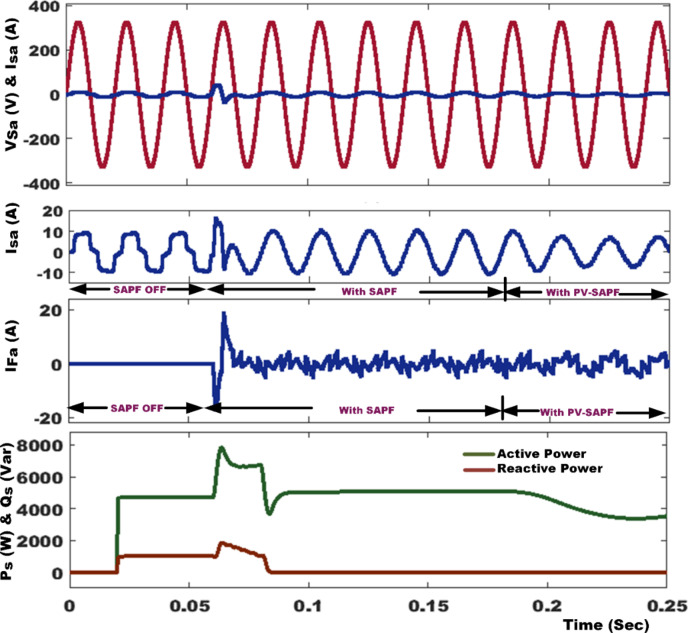
Simulation results of system performance under nonlinear load condition.

**Fig. 10:**
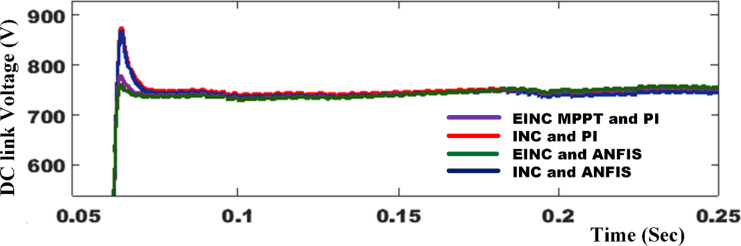
The control of DC-link capacitor utilizing various control techniques.

**Fig. 11:**
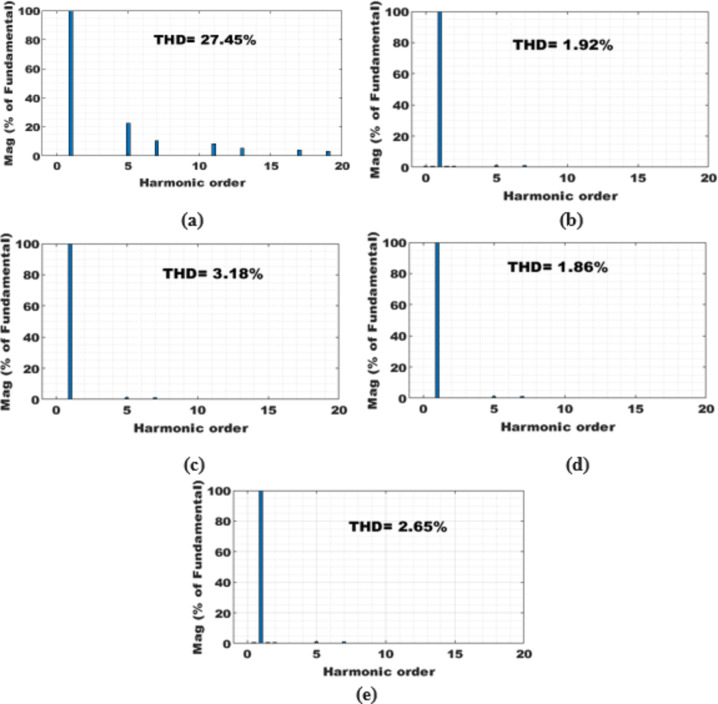
Harmonic spectrum of under NLs: (**a**) Before compensation of source current, (**b**) after compensation of source current using DPC, (**c**) After compensation of PV with SAPF of source current using DPC, (**d**) After compensation of source current using PDPC, (**f**) After compensation of PV with SAPF of source current using PDPC.

Figure 9 provides a comprehensive analysis of the source voltage (V_sa_) and current (I_sa_) before and after compensating for the NLs in both SAPF mode and PV with SAPF mode of operation. The analysis also encompasses the filter current (I_Fa_), active power (P_s_), and reactive power (Q_s_). In the absence of the SAPF, the source and load currents exhibit similar characteristics, with the existing NLs generating a harmonic current that results in a THD of 27.45%. However, when the SAPF is activated at t = 0.06 s, a harmonic current is introduced in the opposite direction to reduce the harmonics in the source current and also compensates for the load’s reactive power demand from the source, achieving a unity power factor, the %THD is mitigated to 1.86% using PDPC technique. We conduct a quantitative analysis of the average power flow at the PCC under these specific conditions, both before and after compensation. It is essential to ensure that the DC link voltage aligns precisely with its established reference value, as highlighted in Fig. 10; Table 4 provides a detailed performance assessment of the DC link voltage within the SAPF under various control algorithms. The PV system is activated at 0.18 s; this condition is recognized as real power injection with SAPF mode. In this mode, PV-SAPF compensates for the load’s harmonic current, supports the reactive power demand, and enhances the overall PQ of the grid. Under nonlinear loads, Fig. 11 illustrates the harmonic spectrum: (a) the THD is 27.45% before source current compensation; (b) it is reduced to 1.92% after source current compensation using DPC; (c) it becomes 3.18% following PV compensation with SAPF alongside source current compensation using DPC; (d) after employing PDPC for source current, the THD is 1.86%, and (e) after compensating the PV with SAPF and applying PDPC to the source current, the THD is 2.65%.

**Table 4:** Performance of DC link voltage in SAPF with different control algorithms.

Case	Before compensation	After compensation	
		PI Controller	ANFIS controller
Maximum overshoot (volt)	--	853	764
Transient settling time (sec)	--	0.072	0.066
% THD	27.45	1.86	1.62

In the context of variable load conditions, the PV-SAPF commenced operation at t = 0.18 s under a NLs condition. The PV-SAPF effectively compensated for the nonlinear load by generating harmonic currents, thereby fulfilling the reactive power demands while simultaneously supplying active power from the photovoltaic source. At t = 0.5 s, a linear load was introduced, characterized by a resistance of *R* = 55Ω and an inductance of L = 140 mH. This transition resulted in an increase in the source current, leading to a slight reduction of approximately 6.5 V in the voltage of the DC link capacitor. During this period, the load drew additional energy from the capacitor to meet its power requirements. The application of the PDPC-SVM technique to the SAPF, both prior to and following the load changes, demonstrated that both real and reactive power quantities adhered closely to their reference values, ensuring a prompt return to these values.

**Fig. 12:**
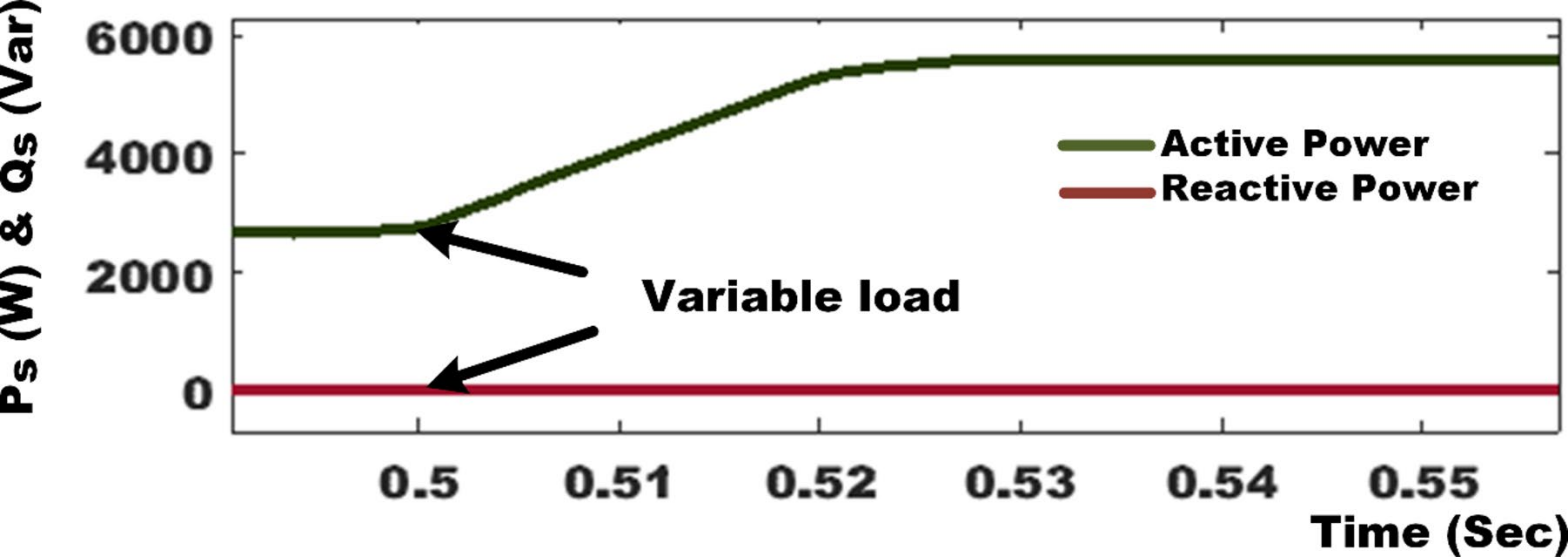
Active and reactive power responses in steady-state and transient conditions.

Subsequently, at t = 0.7 s, the load was further increased to *R* = 110Ω and L = 140 mH, a condition that was sustained until t = 1 s. The ANFIS controller successfully maintained the DC voltage at its reference level of 750 V within a mere 0.03 s. The active and reactive power at the PCC is illustrated in Fig. 12. Under the variable load conditions, reactive power was effectively compensated following the connection of the filter, with the active power aligning perfectly with its reference value.

At t = 1 s, the linear load experienced a reduction, adjusting to a resistance of *R* = 55Ω and L = 140 mH. This load reduction triggered a corresponding decrease in the source current, which resulted in a minor yet significant increase of approximately 4.5 V in the voltage across the DC link capacitor. This voltage change enabled the system to maintain the DC voltage at its reference level of 750 V within a short duration of 0.03 s, facilitated by the DC link controller. Moreover, both the instantaneous active and reactive power remained stable throughout this adjustment period, closely adhering to their assigned reference values. The input currents exhibited nearly sinusoidal waveforms, indicative of effective regulation and synchronization with the source voltages. This optimal performance was achieved by controlling the reactive power to a level near zero.

**Fig. 13:**
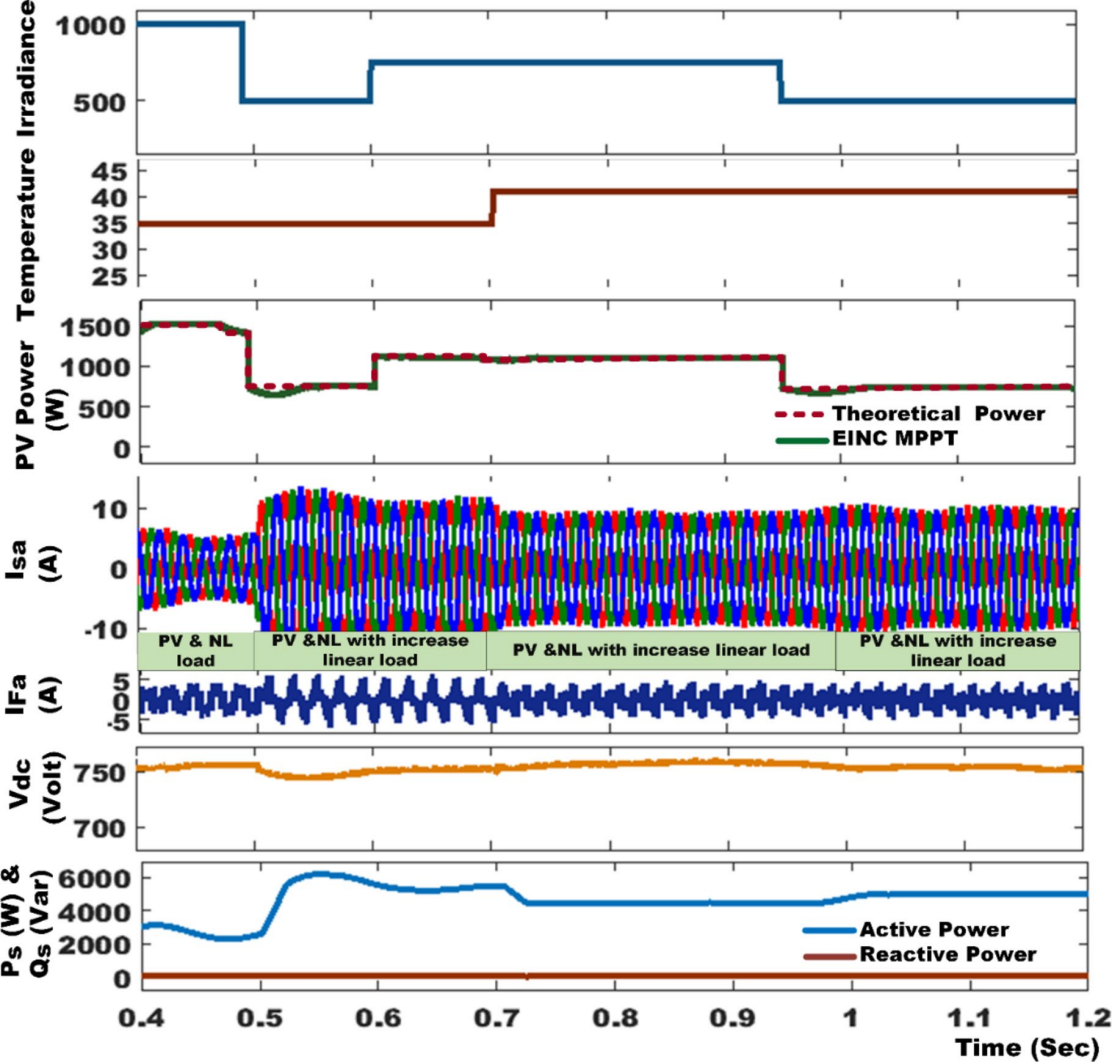
The simulation results reveal SAPF predictive control responses and transient and steady-state active and reactive power responses during robustness test variable load condition.

Figure 13 illustrates the simulation results, showcasing the PV-SAPF predictive control responses, as well as the transient and steady-state responses of active and reactive power under robust testing conditions with variable loads. Table 5 presents a comparative analysis of the system under variable linear with nonlinear load conditions, revealing that in the absence of the SAPF, the source current deviates from a sinusoidal waveform, with THD values as high as 17.17% and 19.79% under variable loads. The integration of the SAPF, governed by either the DPC or PDPC algorithms, markedly enhances the waveform of the source current, enabling conformity with international quality standards, albeit with varying THD values. Notably, with the SAPF regulated by the standard DPC algorithm, the THD is significantly reduced to 1.84% and 2.25%. Conversely, the SAPF filter managed by the PDPC algorithm exhibits an even more substantial reduction in THD, achieving values of 1.42% and 1.51%. This comprehensive analysis underscores the efficacy of the PV-SAPF in maintaining power quality and stability amidst variable load conditions.

**Table 5:** Comparative results analysis of the percentage of source current THD.

Method of Performance		Before Compensation	DPC		PDPC	
			SAPF	PV-SAPF	SAPF	PV-SAPF
%THD of Source Current	Nonlinear load	27.45	1.92	3.18	1.62	2.65
	Linear load (*R* = 55 Ω and L = 140mH) with NLs	17.17	1.84	2.68	1.42	2.23
	Linear load (*R* = 110 Ω and L = 140mH) with NLs	19.79	2.25	2.48	1.51	1.68

## Experimental results and analysis

**Fig. 14:**
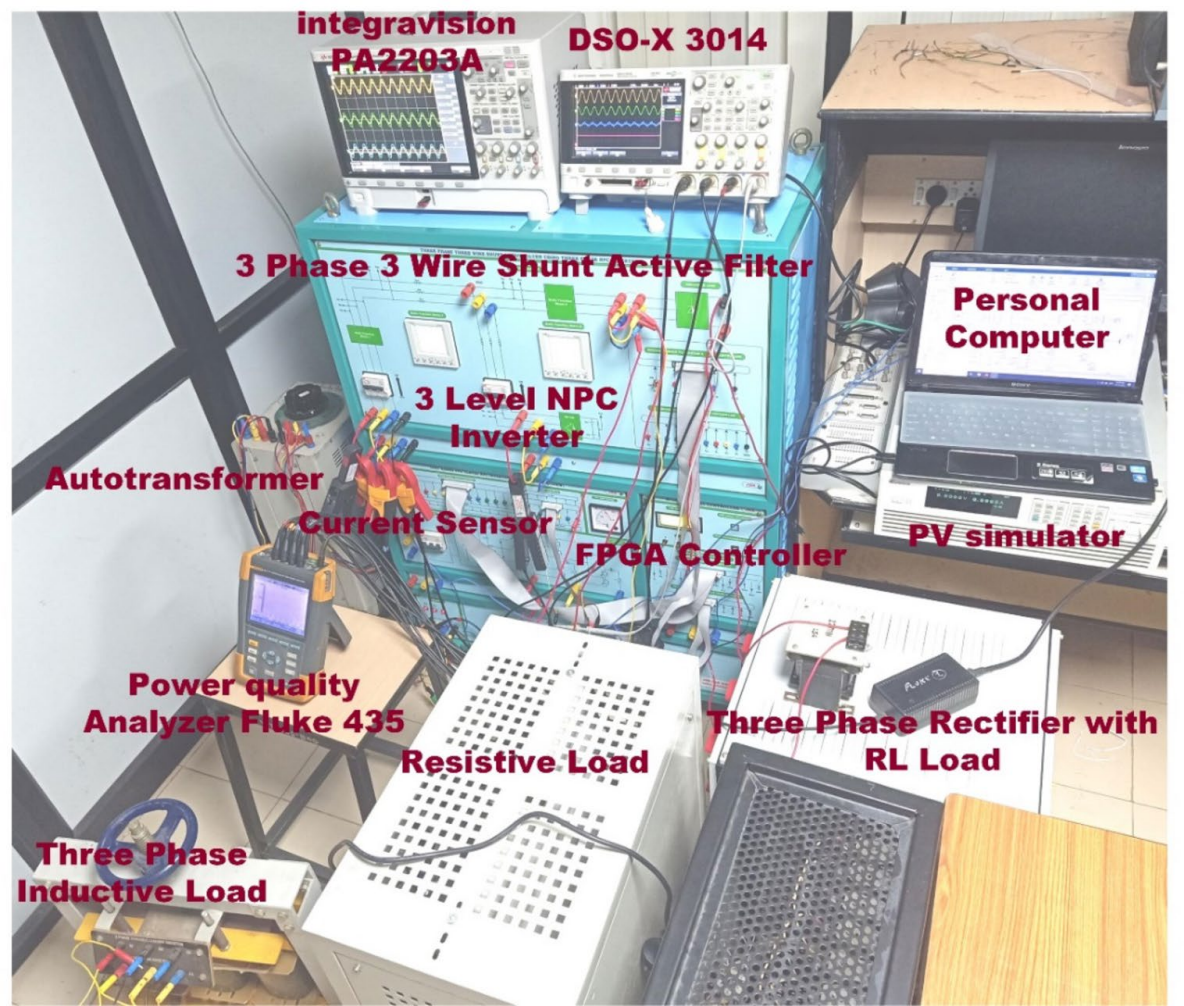
The SAPF was implemented using the SRF technique on the laboratory’s experimental setup.

This section delineates the empirical findings, substantiating the viability and efficacy of the proposed PDPC and SVM methodology for a 3 L SAPF. We implemented the control algorithm using the Vivado 2023.2-Artix-7 DSP FPGA controller, which executes a PDPC-SVM-based algorithm for the SAPF system. Table 6 contains the parameters of the algorithm. Figure 14 displays a photograph of the experimental setup, which represents the SAPF prototype model.

### FPGA implementation of PDPC theory

**Fig. 15:**
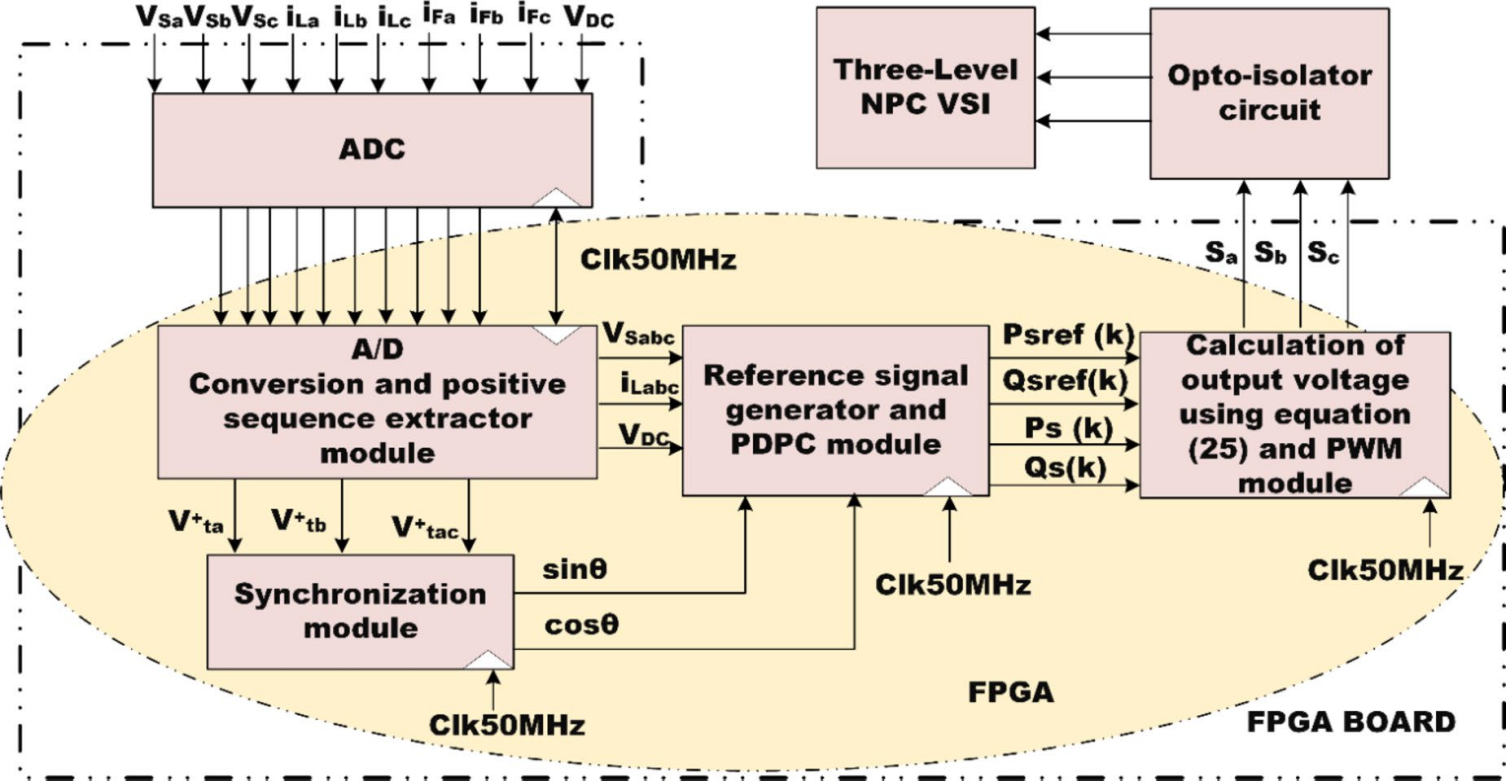
FPGA implementation of comprehensive design and operational capabilities.

The measurement of load voltages and currents for each phase is accomplished using the Hall Effect Current Sensor-HE100T01 and the Voltage Sensor-IC 7840. These transducers not only provide essential measurement capabilities but also create electrical isolation between the control circuitry and the power circuits. To ensure compatibility with the analog channels of the FPGA, the sensor signals undergo a signal conditioning process, converting them into a standardized range of 0 to 5 V. The outputs from these conditioning circuits are interfaced with 12-bit A/D converters-IC AD7366, and the resulting digital data is transmitted to the FPGA processor via the IO lines. The FPGA program generates switching signals for IGBTs based on the principles of PDPC. A robust driver circuit-TLP 250 IC is crucial for maintaining the minimum current requirements necessary for optimal driver operation. Specifically, four driver circuits are deployed to provide switching pulses for the IGBT modules-SKM100GB12T4. The implementation of the SAPF utilizes the SEMITRANS 2 Fast IGBT Module, which features a three-level diode clamped inverter architecture. The configuration and design of the SAPF prototype model are illustrated in Fig. 15, highlighting the system’s comprehensive design and operational capabilities.

**Table 6:** Validation experimental design parameters.

Components	Parameter	Value
Grid	Source voltage (Phase voltage)	230 V,50 Hz
Filter	Inductance	5 mH
DC-link voltage	Capacitor	2200µF
Nonlinear load	Resistor and inductor	*R* = 80Ω and L = 12mH
Load	Resistive load	0.5 kW to 2 kW
	Inductive load	140mH
Chromo PV simulator (model-62050 H–600 S)	Open circuit voltage, V_OC_ Short circuit current, I_SC_	600 V 6 A

### Steady-State operation under NLs load

**Fig. 16:**
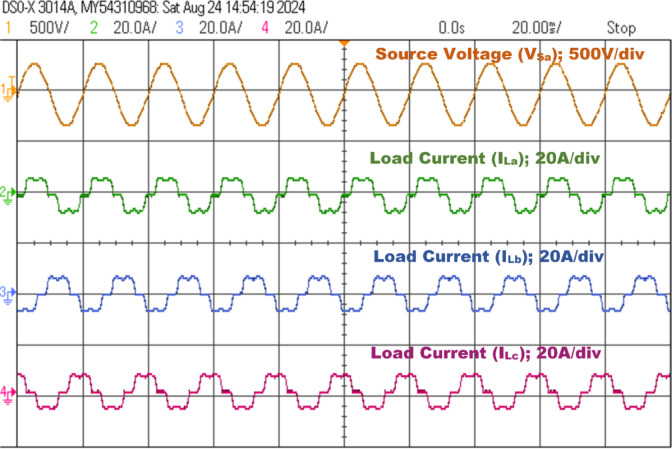
Phase A voltage with RL load with a diode rectifier for a three-phase system and load currents.

**Fig. 17:**
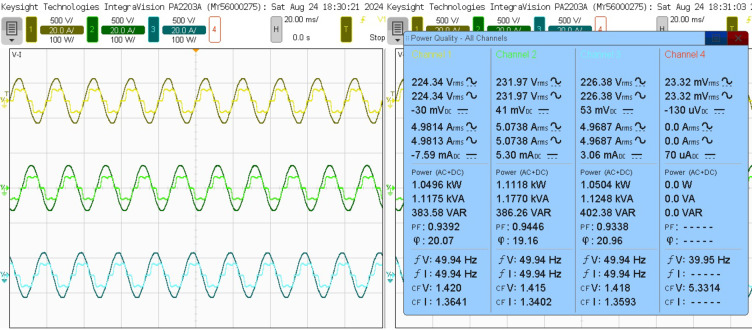
Power quality analysis of the steady-state response before compensation.

This analysis examines the compensating characteristics of a three-phase SAPF, specifically utilizing a 3-Level NPC voltage source inverter controlled by a PDPC implemented with SVM. The focus is on its integration with a diode bridge rectifier connected to an RL load under steady-state conditions. The nonlinear characteristics of the load generate a harmonic current, leading to a non-sinusoidal load current profile. The input source voltage and load current is depicted in Fig. 16. The power quality assessment during this analysis indicates a real power of 1.0496 kW, reactive power of 383.58 VAR, and apparent power of 1.1175 kVA, resulting in a power factor of 0.9398 and a phase angle of 20.07 degrees, as shown in Fig. 17. Furthermore, the source current exhibits both the fundamental component and substantial harmonic components at the PCC, with a THD measured at 25.403%. This level of harmonic distortion exceeds the permissible thresholds established by IEEE 519 standards, as illustrated in Fig. 20(a).

**Fig. 18:**
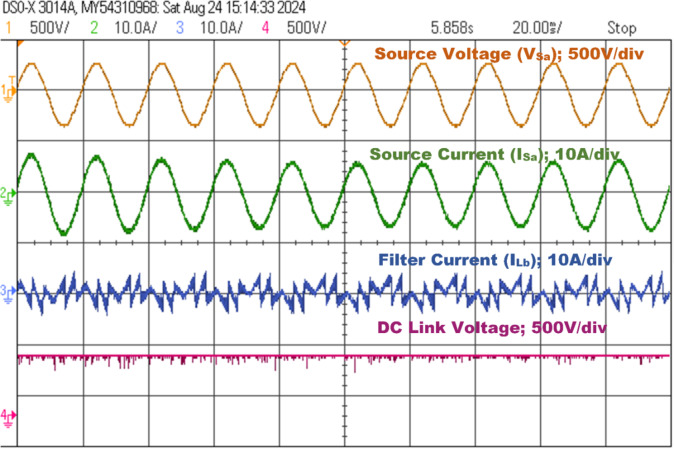
SAPF steady-state response (phase-A).

**Fig. 19:**
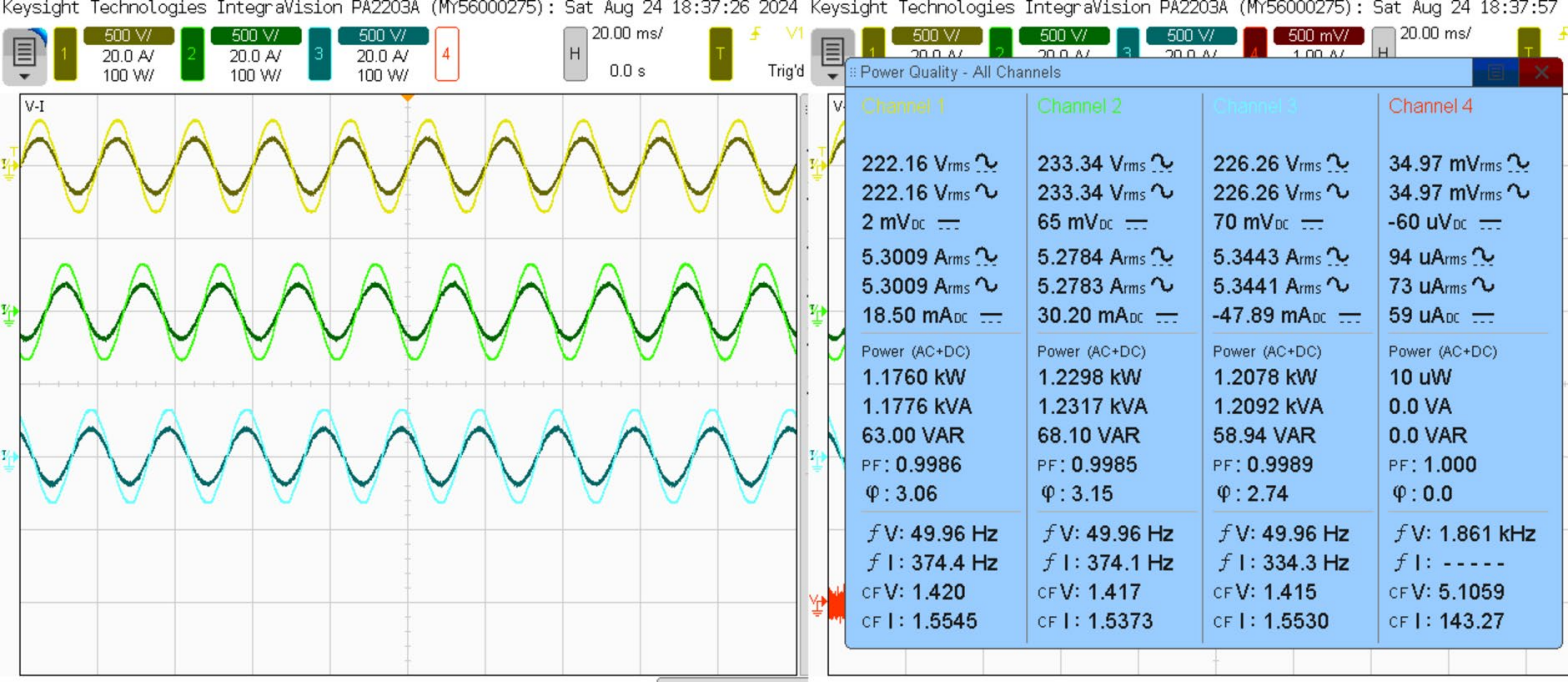
Power quality analysis of the steady-state response after compensation.

**Fig. 20:**
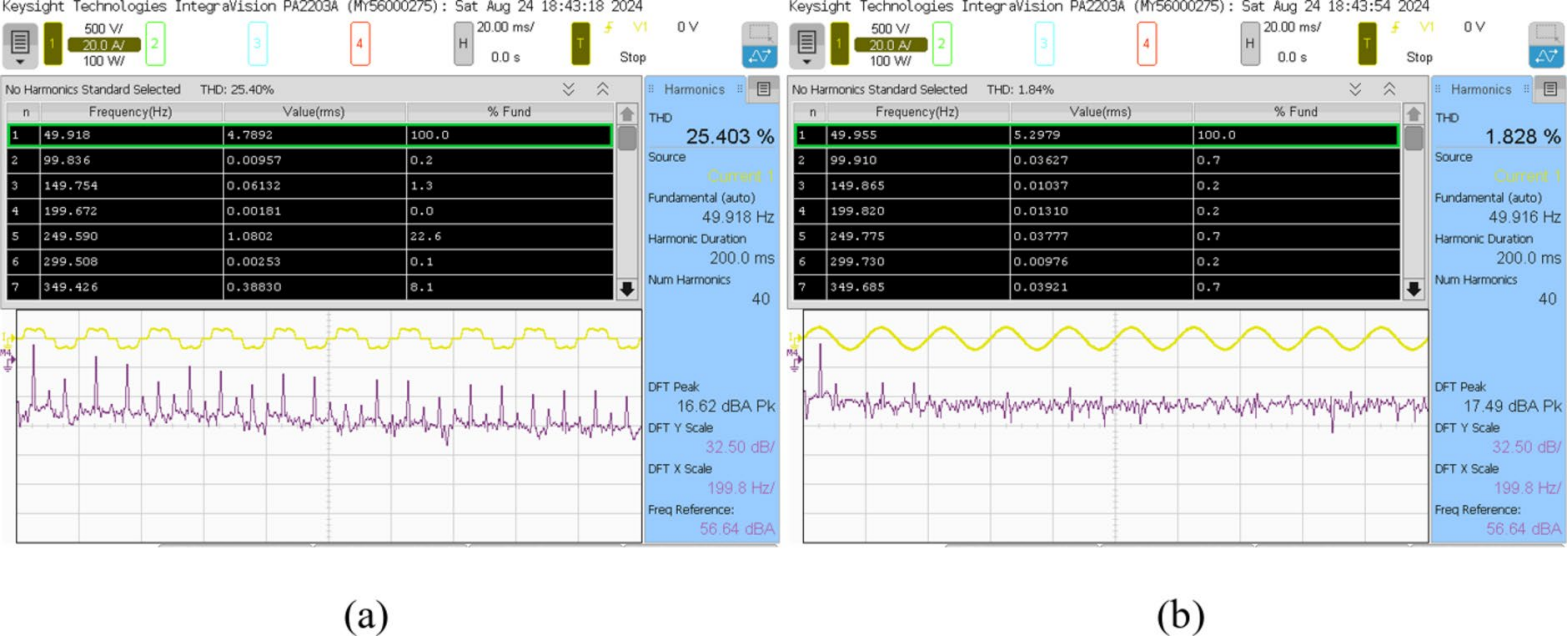
Percentage of total harmonic distortion of source current: (**a**) Before compensation, (**b**) After compensation.

The SAPF must produce a counteracting harmonic current, which is then directly linked to the PCC. The 3 L NPC-based SAPF achieves reactive power compensation, diminishes harmonics in the source current for non-linear loads, and upholds a power factor of one on the source side, Fig. 18 shows the examination of the source voltage, source current, filter current, and DC link voltage in a steady-state analysis phase A after compensation and illustrated in Fig. 19 of the power quality analysis of real, reactive and apparent power, power factor, phase angle. Figure 20 (a) and (b) illustrate the source current prior to and subsequent to compensation, respectively. It is observed that the THD has been significantly mitigated from 25.403 to 1.826%.

### Analysis of the performance of SAPF under varying linear load with NLs load condition

**Fig. 21:**
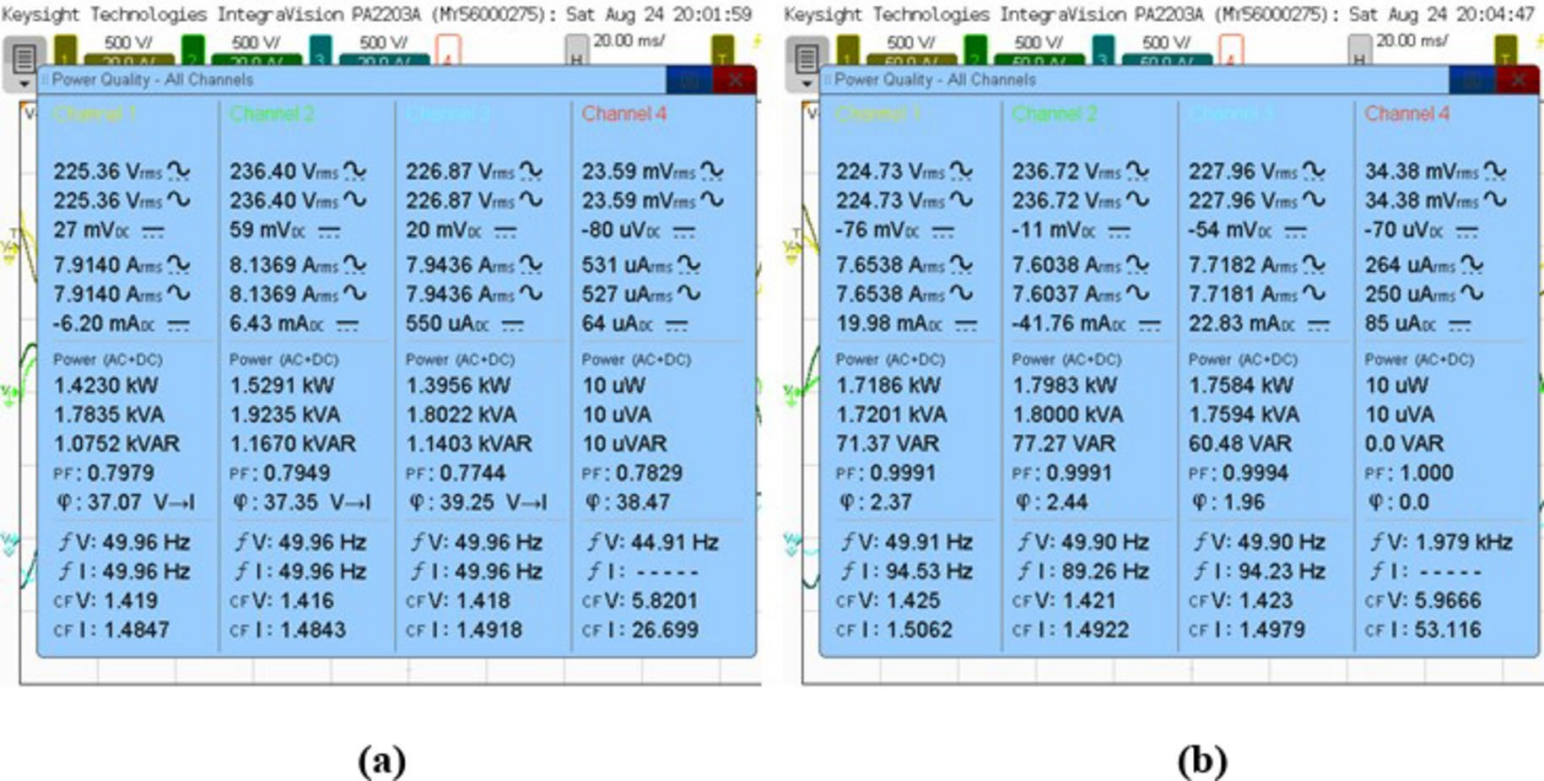
Power quality analysis of the steady-state response: (**a**) Before compensation, (**b**) After compensation.

The conducted analysis delineates the compensation strategies for reactive power and harmonics associated with both parallel linear loads, characterized by a resistance of 0.5 kW and an inductance of 140 mH, and non-linear loads. Within this framework, the efficacy of the SAPF is meticulously assessed. Subsequent to the adjustment phase, it was noted that the power factor on the supply side experienced a substantial improvement, escalating from 0.79 to 0.99 lag, as corroborated by the empirical data presented in Fig. 21 (a) and (b). Concurrently, there was a pronounced reduction in the supply reactive power, which diminished from 1.0752 kVAR to 71.32 VAR.

**Fig. 22:**
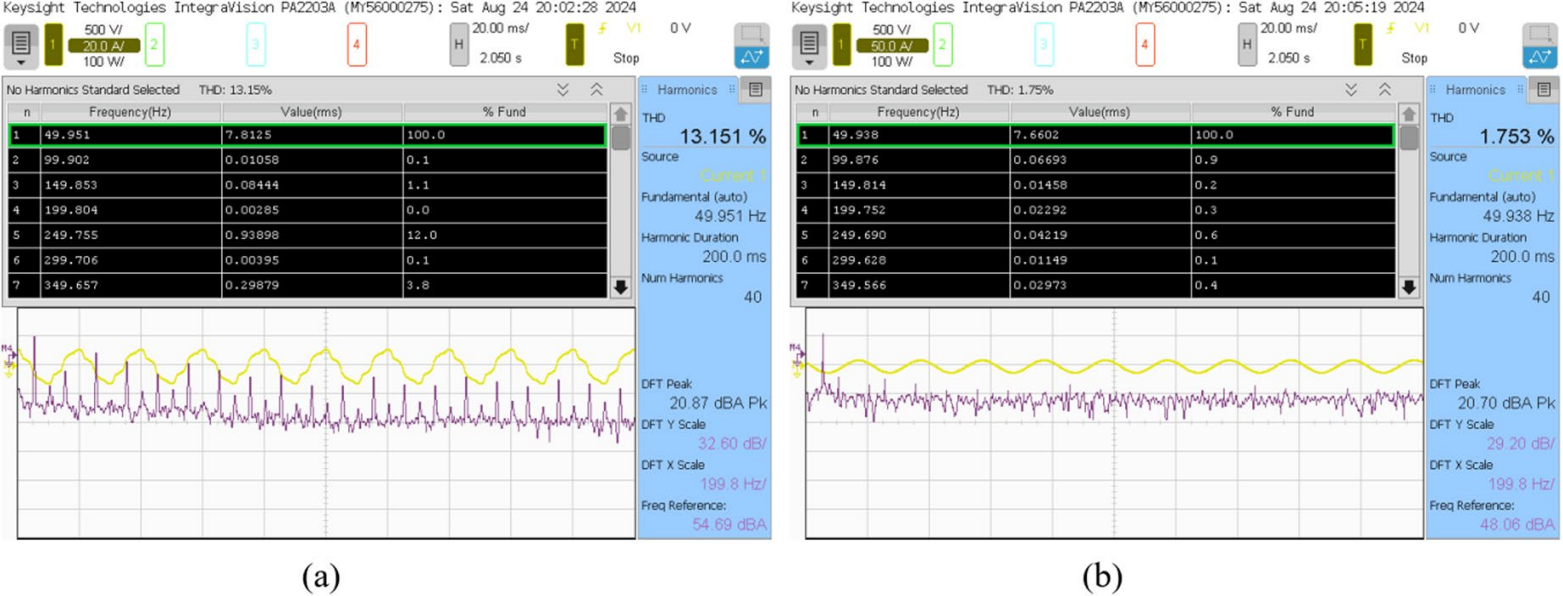
Percentage of THD of source current: (**a**) Before compensation (**b**) After compensation.

The research investigated various configurations with inductance values of 140 mH and resistance levels ranging from 0.5 kW to 2 kW. The goal was to evaluate the effectiveness of SAPF in improving system characteristics through a comprehensive analysis. The study found that the injected current precisely tracked the reference current, even when there were fluctuations in the load conditions. Additionally, there was a notable reduction in the reactive power supplied by the source and an improvement in the power factor. This indicates that the recommended approach performs optimally. The findings are succinctly outlined in Table 7.

**Table 7:** Performance of varying linear load condition.

		Inductive Load L = 140mH with NLs load			
		*R*_L_=0.5 kW	*R*_L_=1 kW	*R*_L_=1.5 kW	*R*_L_=2 kW
Before compensation of SAPF	P (kW)	1.4230	1.7803	2.1150	2.5465
	S (kVA)	1.7835	2.1019	2.4141	2.8512
	Q (kVAR)	1.0752	1.1175	1.1641	1.2825
	PF	0.7979	0.8470	0.8761	0.8931
	φ	37.07	32.12	28.83	26.73
	% of THD	13.151	11.071	9.770	8.696
After compensation of SAPF	P (kW)	1.7186	2.1516	2.6151	3.0193
	S (kVA)	1.7201	2.1535	2.6173	30,208
	Q (Var)	71.37	−89.92	−108.62	−95.26
	PF	0.9991	0.9991	0.9991	0.9995
	φ	2.37	2.39	2.38	1.81
	% of THD	1.753	1.670	1.622	1.406

P = Active power; Q = Reactive power; S = Apparent power; PF = Power factor;

### Analysis of the SAPF’s performance under dynamic loading conditions

**Fig. 23:**
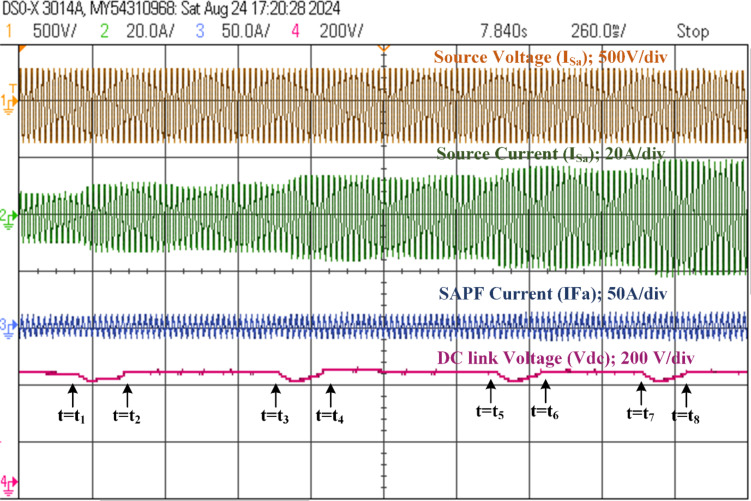
Dynamic loading of change from 0.5 kW to 2 kW load condition response of SAPF (Phase-A).

The investigation focuses on the dynamic responsiveness of a developed FPGA-based SAPF system in the context of sudden load fluctuations under nonlinear load conditions. Initially, the SAPF is engaged to compensate for the nonlinear load, generating the harmonic current and reactive power necessary for stability. At time t_1_, a linear load is introduced, resulting in an increase of 0.5 kW. The system’s DC link voltage successfully reaches a steady-state condition by time t_2_, stabilizing within twenty milliseconds. This steady state is maintained from t_2_ to t_3_. As the demand grows further, the load is increased from 0.5 kW to 1 kW at t_3_, with the system once again achieving a steady-state response by t_4_. This pattern continues as the load is incremented from 1 kW to 1.5 kW at t_5_, reaching stability by t_6_, and then from 1.5 kW to 2 kW at t_7_, ultimately stabilizing by t_8_. The intervals t_1_-t_2_, t_3_-t_4_, t_5_-t_6_, and t_7_-t_8_, represent the transient phases, while the segments between t_2_-t_3_, t_4_-t_5_, and t_6_-t_7_ denote the corresponding durations of steady-state operation, as illustrated in Fig. 23. Additionally, Table 7 presents the performance metrics under various linear load conditions, detailing the active and reactive power delivered by the source both prior to and following compensation. The effective regulation of the DC-link voltages is critical for maintaining a stable voltage level, which in turn enables the generation of sinusoidal and balanced source currents. This stabilization of the current waveform significantly enhances the stability of the fundamental frequency, particularly in scenarios characterized by fluctuating loads. Moreover, the voltages across the DC capacitors remain balanced even in the presence of variations in both active and reactive power, underscoring the robustness of the compensation mechanism.

The thorough comparative analysis of the PDPC strategy about various innovative control methodologies identified in the existing literature demonstrates a significant advancement in understanding their relative effectiveness. Our evaluation, based on the THD derived from real-time applications utilizing the diverse algorithms and control techniques outlined in Table 8, reveals that the PDPC combined with SVM and ANFIS controllers for the SAPF achieves superior performance, particularly in source current total harmonic distortion. The empirical results distinctly highlight the advantages of our approach in enhancing system efficiency and reliability.

**Table 8:** Comparative analysis of the proposed control strategies with the recent studies.

Control strategy		(*p*–q) method based on parameters^41^	Direct method based on modified SRF proposed in^42^	DPC strategy proposed in^43^	Predictive control strategy proposed in^44^	Predictive control strategy proposed in^45^	PDPC with SVM strategy and ANFIS controller
Before Compensation of source THD current	Simulation	20.03%	57.4%	27.48%	---	22.60%	27.45%
	Experiment	24.65%	70.3%	23.4%	29%	23.6%	25.403%
After Compensation of source THD current	Simulation	1.78%	0.3%	0.91%	---	0.81%	1.62%
	Experiment	5.4%	4.2%	5.5%	5.56%	3.12%	1.828%
Main features		Fixed switching frequency, High number of sensors, High complexity.	Fixed switching frequency, Reduced number of sensors, High complexity.	Variable switching frequency, Reduced number of sensors, Ease implementation.	Fixed switching frequency, High number of sensors, Ease implementation.	Variable switching frequency, Reduced number of sensors, Ease implementation.	Fixed switching frequency, Reduced number of sensors, Ease implementation.

### Performance of PV integrated with SAPF under NLs load

Initially, the PV output power was not operational, and the grid was the supplier of power to the diode bridge RL load. Figures 16, 17 and 18, and 19 illustrate the transfer of total actual power from the power source to the load during operation. In this scenario, only the SAPF compensates for the reactive power demand of NLs by generating harmonic current. The total harmonic distortion has decreased from 25.403 to 1.826%, and the current from the power supply has increased from 4.98 to 5.3 Amperes.

**Fig. 24:**
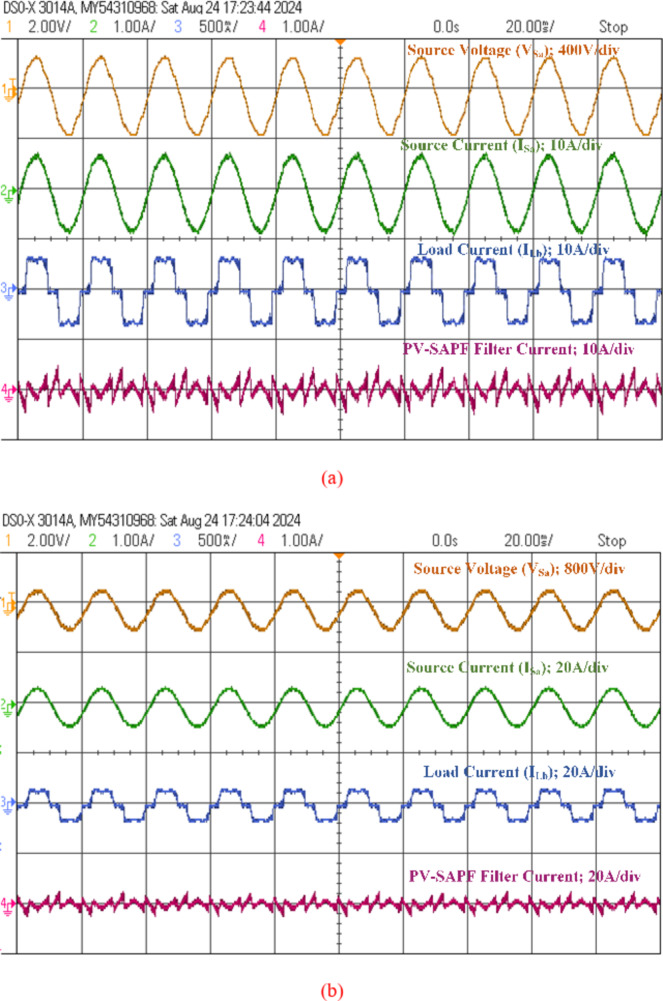
Analysis of PV Integration with SAPF: (**a**) scenario1: PV System Providing a Net Output Power of 1200 Watts to the Load from the PCC. (**b**) Scenario 2: PV System Delivering a Net Output Power of 1500 Watts to Both the Grid and Load.

In the second scenario, both the boost converter and NPC inverter are activated to receive PV power. The current flowing through the converter consistently maintains its predefined magnitude, while the voltage across the DC-link capacitor (V_dc_) converges towards the designated target value. The photovoltaic system is connected to the SAPF to deliver active power from the PV-SAPF to the load. During steady-state operation, the dual-purpose inverter transfers real power from the PV array, providing 1200 watts of power. Active power delivery from the grid is reduced from 3658.94 watts to 2459.65 watts at the PCC. Similarly, reactive power delivery from the grid is reduced from 194.47 var to 134.07 var, and it also provides compensation for reactive power demand and harmonic components of the load current, ensuring synchronization of the grid current with the voltage at the PCC. Figure 24 (a) illustrates the grid voltage, grid current, load current, and compensating filter current. At the same time, PV power increased from 1200 watts to 1500 watts, as shown in Fig. 24(b). Comparison of Table 9 shows source power, phase angle, power factor, and percentage of THD under SAPF and PV-SAPF conditions using experimental testing.

**Table 9:** Comparison of power analysis of before and after compensation of SAPF and PV-SAPF conditions using experimental testing.

	Source power				φ	Power factor	% THD
	V_ph_ (V)	I_ph_ (A)	*P*_total_ (W)	Q_total_ (VAR)			
SAPF OFF State	230	5.0	3211.8	1172.22	20.07	0.93	25.403
SAPF ON State	230	5.31	3658.94	190.47	2.98	0.99	1.828
PV-SAPF (1200 W)	230	3.57	2459.65	134.07	3.12	0.99	2.28
PV-SAPF (1500 W)	230	3.14	2163.24	120.56	3.19	0.99	2.16
PV-SAPF (1800 W)	230	2.7	1861.05	85.16	3.14	0.99	2.08

## Conclusion

This paper presents a real-time implementation of photovoltaic systems integrating with a three-phase NPC-based shunt active power filter. The SAPF uses PDPC techniques to compensate for harmonics and reactive power. The algorithm has been validated in a practical laboratory environment. The predictive approach is found to be more efficient and superior to the typical DPC command for the SAPF function. This sophisticated technique ensures accurate and efficient control of both active and reactive power, provides a sinusoidal source current for the power supply, and improves the power factor, approaching unity. The implementation of ANFIS approaches for regulating DC-link capacitor voltage have significantly enhanced system robustness, diminished the vulnerability to system transient phenomena, minimal steady-state errors, and low power ripple, and ensured the stability of the DC bus. The effectiveness of the 3 L active power filter has been assessed across various load conditions, and simulation and hardware results confirm its robustness for accurate reference tracking, achieving high-quality grid current with low total harmonic distortion in compliance with IEEE-519 standards and a power factor of unity. The system under investigation has significantly improved its performance, offering potential for energy efficiency improvements in the industrial sector. The predictive SAPF is being extended to renewable energy, improving energy quality from eco-friendly sources, prolonging equipment lifespan, and contributing to environmental preservation. The experimental findings indicate that the THD is 25.403% before compensation, which decreases to 1.828% after compensation for NLs loads. When the system is operating under linear load conditions with *R* = 0.5 kW and L = 140mH, the THD is measured at 13.151% before compensation and 1.753% after compensation. The system’s performance under dynamic load conditions is also examined, yielding satisfactory results. Furthermore, the study delves into active power generation using PV systems under various supply conditions. Analysis has shown that the PV integrated with the SAPF system demonstrates outstanding performance improvements. These include enhanced reactive power compensation and power factor, reduced source current THD and minimized fluctuations in DC-link voltage, smoothed the load demand curve and eliminated the need for an additional grid-tied inverter.

## Data Availability

The datasets used and/or analysed during the current study available from the corresponding author on reasonable request.
